# Utilising systematic reviews to assess potential overtreatment and claim for better evidence-based research: an analysis of anticancer drugs versus supportive care in advanced esophageal cancer

**DOI:** 10.1186/s13643-024-02594-1

**Published:** 2024-07-18

**Authors:** Marilina Santero, Adriana-Gabriela Meade, Anna Selva, Olga Savall-Esteve, Javier Bracchiglione, Ismael Macías, Leire Leache, Paula Cerdà, Xavier Bonfill Cosp, Appropriateness of Systemic Oncological Treatments for Advanced Cancer (ASTAC-Study) Research Group, Roberto Acosta-Dighero, Alba Antequera, Ariadna Auladell-Rispau, Yahveth Cantero-Fortiz, Edgar D Hernández, Juan Irassar, Pamela Meinardi, Angela Merchán-Galvis, Nicolas Meza, María Jesús Quintana, Carolina Requeijo, Gerardo Rodríguez-Grijalva, Karla Salas-Gama, Josefina Salazar, Olga Savall-Esteve, Ivan Solà, Gerard Urrútia

**Affiliations:** 1 Universitat Autònoma Barcelona, Barcelona, Spain; 2 Iberoamerican Cochrane Centre, Institut de Recerca Sant Pau (IR, SANT PAU), Barcelona, Spain; 3 Clinical Epidemiology and Cancer Screening, Parc Taulí Hospital Universitari, Institut d’Investigació I Innovació Parc Taulí (I3PT_CERCA), Sabadell, Spain; 4 Interdisciplinary Centre for Health Studies (CIESAL), Universidad de Valparaíso, Viña del Mar, Chile; 5 CIBER de Epidemiología y Salud Pública (CIBERESP), Barcelona, Spain; 6 Servicio Oncología Médica, Hospital de Sabadell-Corporació Sanitària Parc Taulí, Sabadell, Barcelona, Spain; 7 Unit of Innovation and Organization, Navarre Health Service, Pamplona, Spain; 8 Navarra Institute for Health Research (IdiSNA), Pamplona, Spain; 9 Hospital de La Santa Creu I Sant Pau, Barcelona, Spain

**Keywords:** Advanced esophageal cancer, Chemotherapy, Targeted therapy, Immunotherapy, Systematic review, Meta-analysis

## Abstract

**Background:**

Highlighting the identified gaps in evidence-based research concerning advanced esophageal cancer (EC) treatment and care, this review evaluates the efficacy and safety of anticancer drugs compared to supportive care for advanced EC patients, aiming to assess the appropriateness of usual treatments and identify the gaps that need to be filled with primary research.

**Methods:**

We searched (May 2022) MEDLINE, EMBASE, the Cochrane Central Register of Controlled Trials (CENTRAL), Epistemonikos, and trial registries (ClinicalTrials.gov and PROSPERO) for randomised controlled trials (RCTs) comparing anticancer drugs (chemotherapy, immunotherapy, or biological/targeted therapy) with supportive care in advanced EC. The results were summarised using GRADE summary of finding tables.

**Results:**

We included 15 RCTs. Most studies did not have a special focus on EC, did not detail the treatment lines in all patients, and did not evaluate all outcomes. Anticancer drugs may result in a slight increase in overall survival (OS) (HR 0.78; 95% CI 0.71, 0.86; MD 0.83 months) and better progression-free survival (PFS) (HR 0.56 95% CI 0.49, 0.64, MD 0.68 months), but also may increase toxicity (RR 1.37; 95% CI 1.13, 1.65), without a significant improvement in quality of life. The certainty of evidence was low or very low due to indirectness of results and lack of specific focus on EC in some studies.

**Conclusion:**

RCTs on advanced EC lack specificity, detailed treatment line information, and evaluation of all relevant outcomes. Moreover, when they find any benefit, this is negligible. Therefore, the certainty to justify anticancer drug treatments instead of supportive care in advanced EC is low or very low, and this information should be actively shared with affected patients. More and better RCTs should be conducted to assess whether any old or new proposed treatment for advanced EC patients provides a better balance of benefits and harms than the supportive care.

**Systematic review registration:**

The study protocol was registered in OSF (https://doi.org/10.17605/OSF.IO/7CHX6) on 2022–03-29.

**Supplementary Information:**

The online version contains supplementary material available at 10.1186/s13643-024-02594-1.

## Background

Esophageal cancer (EC) is a worldwide public health problem and a leading cause of death [1, 2]. It has been estimated that 39.0% of patients are diagnosed at an advanced stage, resulting in a very poor survival rate, even in high-income countries [1]. Likewise, age-standardised 3-year net survival rates for advanced stages range from 4.4 to 7.4% [3]. 

Over the last two decades, a spectrum of single or combined anticancer drugs has shown improvement in response rates [4]. However, outcomes are still unsatisfactory due to the limited efficacy and severe adverse effects of conventional treatments [5]. As a consequence, new systemic therapies such as immunotherapy and targeted therapies have emerged, offering promise in extending survival rates. Nonetheless, their efficacy is still limited, and their use is associated with a high incidence of adverse events and toxicities [6–9]. Among them, nivolumab and pembrolizumab in combination with chemotherapy have been introduced to control tumour growth and improve survival. In 2021 and 2022, the Food and Drug Administration (FDA) and the European Medicine Agency (EMA) approved various drug combinations for some patients with advanced EC who are not candidates for surgery, based on the results of the ATTRACTION-3 [10], KEYNOTE-590 [11], and CheckMate 648 trials [12].

Moreover, some patients with advanced EC receive supportive treatments—commonly referred to as supportive care—to improve symptom control and quality of life (QoL). Supportive care encompasses a spectrum of interventions targeting symptom control, treatment side effects, and patient well-being during cancer therapy. These measures may include pain management, nutritional support, psychosocial assistance, and palliative care [13] either as a unique treatment or combined with anticancer drugs [14–16]. There are lingering uncertainties regarding whether these two approaches can be effectively combined and utilised together for patients, as well as questions surrounding the potential advantages and disadvantages of their combined application [17]. Nevertheless, according to our recent overview of systematic reviews (SR) on this topic, the real benefit of anticancer drugs compared to any supportive approach in patients with advanced EC remains uncertain and has been inadequately summarised [18]. Thus, the optimal therapy for patients with advanced EC is still debatable.

Therefore, patients with advanced EC represent a real challenge from a clinical perspective, due to the short life expectancy and the difficulty of balancing the potential benefit of offering anticancer drugs with their associated side effects and related costs. The lack of confidence in evidence may hinder decision-making in clinical practice and is a crucial issue of end-of-life care. Consequently, this review aims to assess all the available evidence on the efficacy and safety of anticancer drugs compared to supportive care in patients with advanced EC.

## Methods

### Study design

We developed a SR according to the Cochrane Handbook for Systematic Reviews [19, 20], and reported it following the PRISMA 2020 statement [20]. This SR is part of a wider project (ASTAC-Study) aiming to conduct broad evidence syntheses assessing the effects of systemic anticancer drugs compared to supportive care for people with advanced non-intestinal digestive cancers [18, 21–23]. The review protocol was prospectively published on March 29, 2022, in Open Science Framework (OSF) [21].

### Eligibility criteria

Studies had to meet all the following criteria of eligibility (details are presented in Additional file 1): (1) randomised controlled trials (RCTs); (2) included adult patients diagnosed with advanced or metastatic, primary or recurrent carcinoma of the esophagus or gastroesophageal junction (GEJ); (3) systemic anticancer drugs as intervention (chemotherapy, immunotherapy, or biological/targeted therapy); (4) any supportive treatment as a comparison (supportive care, observation, or placebo).

Advanced EC corresponds to stages IIIb, IIIc, or IV [18]. The authors may refer to as “unlikely to be cured” or they may also use the terms “secondary”, “metastatic”, “terminal”, “advanced”, or “progressive” cancer. We included studies involving only a subset of eligible participants (for example studies including participants with both GEJ and gastric cancer) if they provided disaggregated data for GEJ cancer and included at least 15 eligible participants.

The exclusion criteria include at least one of the following: (1) neuroendocrine, stromal, or lymphatic neoplasms; (2) surgery or radiotherapy as sole interventions; (3) adjuvant or neoadjuvant chemotherapy; (4) non-palliative treatments; (5) quasi-experimental studies, observational studies, reviews, and protocols.

### Search strategy

As part of the wider ASTAC-Study, we conducted a sensitive search strategy in MEDLINE (access via PubMed), EMBASE (access via OVID), the Cochrane Central Register of Controlled Trials (CENTRAL), and Epistemonikos from inception until December 2019, involving all cancer locations assessed. We also searched study registries (ClinicalTrials.gov and PROSPERO). For this specific SR, we updated the search strategy in MEDLINE and CENTRAL until May 2022, using more specific search strings related to advanced EC, and a search filter for RCT. Additional file 2 provides the details of the original electronic search strategy and the updated one. To complement our search, we also conducted a forward and backward citation search strategy using *citation chaser* [24], an R package including a Shiny app for conducting citation chasing from a starting set of articles.

### Study selection and data extraction

Two reviewers (MA, SA, SO, or BJ) independently screened each title and abstract of the references obtained from the search. A third reviewer (SM or LL) resolved any disagreement. Subsequently, the same team of reviewers (MA, SA, SO, or BJ) independently screened the full texts, with any disagreements again resolved by a third reviewer (SM or LL). The reviewers who screened titles and abstracts were not necessarily the same ones who screened the full texts. Before commencing the screening, a pilot test was conducted on a set of references to ensure consistency and refine the selection criteria. For this entire process, *Covidence*, a web-based collaboration software platform, was used [25].

The efficacy outcomes of interest were overall survival (OS) and progression-free survival (PFS). The safety outcome of interest was toxicity measured as the incidence of grade 3–5 adverse events (AEs), according to the Common Terminology Criteria for Adverse Events (CTCAE) grading system. Other outcomes of interest were as follows: performance status (PS), symptoms related to the disease, quality of life (QoL), admissions to a hospital or long-term centre or emergency consultations, and quality of end-of-life care.

Six reviewers were involved in data extraction (SM, MA, SA, SO, BJ, and LL). Each included study was extracted by a randomly assigned pair of reviewers to ensure accuracy. One reviewer extracted data from the included studies using a previously piloted data extraction sheet, and a second reviewer cross-checked this process. Discrepancies were solved by a discussion with an arbiter (SM).

### Risk of *bias* assessment and certainty of evidence

Pairs of independent reviewers assessed the risk of bias (RoB) using the Cochrane RoB tool [26], and each RCT was rated as low, high, or unclear. The RoB across studies was assessed considering the distribution of each RoB domain of the body of evidence included (illustrated in the RoB graph by domain). We estimated the certainty of evidence according to the Grading of Recommendations Assessment, Development, and Evaluation (GRADE) approach [27, 28] for the selected outcomes, which was presented in “Summary of Findings” (SoF) tables. We downgraded the evidence from high quality by one level for serious (or by two levels for very serious) study limitations (RoB), indirectness of evidence, inconsistency, imprecision of effect estimates, or potential publication bias. We classified the certainty of the evidence for each outcome as high, moderate, low, or very low.

### Statistical analysis

For time-to-event outcomes, we extracted hazard ratios (HR) with 95% confidence intervals (95% CI), and the number of events or *p*-values to calculate the logHR and standard error based on intention-to-treat study populations [29]. For dichotomous outcomes, we used the event rates and sample sizes to calculate risk ratios (RR). If the number of events was zero in a treatment arm, we followed guidance provided by the Cochrane Handbook in Sect. 10.4.4 [26]. For continuous outcomes, we calculated the mean difference (MD) or standardised mean difference (SMD). All measures were calculated with their associated 95% CI.

We performed meta-analyses using a random-effects model and inverse-variance weighting for studies only when studies were reasonably homogeneous (both clinically and methodologically). We performed a subgroup analysis according to the specific types of anticancer drugs (chemotherapy, immunotherapy, and biological/targeted therapy).

In the case of statistical heterogeneity, as tested both visually and using the I^2^ index, we explored baseline characteristics of studies and conducted sensitivity analyses. Statistical significance was defined as a *p*-value < 0.05. We used the software Review Manager (RevMan) 5.4.1, provided by the Cochrane Library for all statistical analysis [30].

## Results

### Study selection

**Fig. 1:**
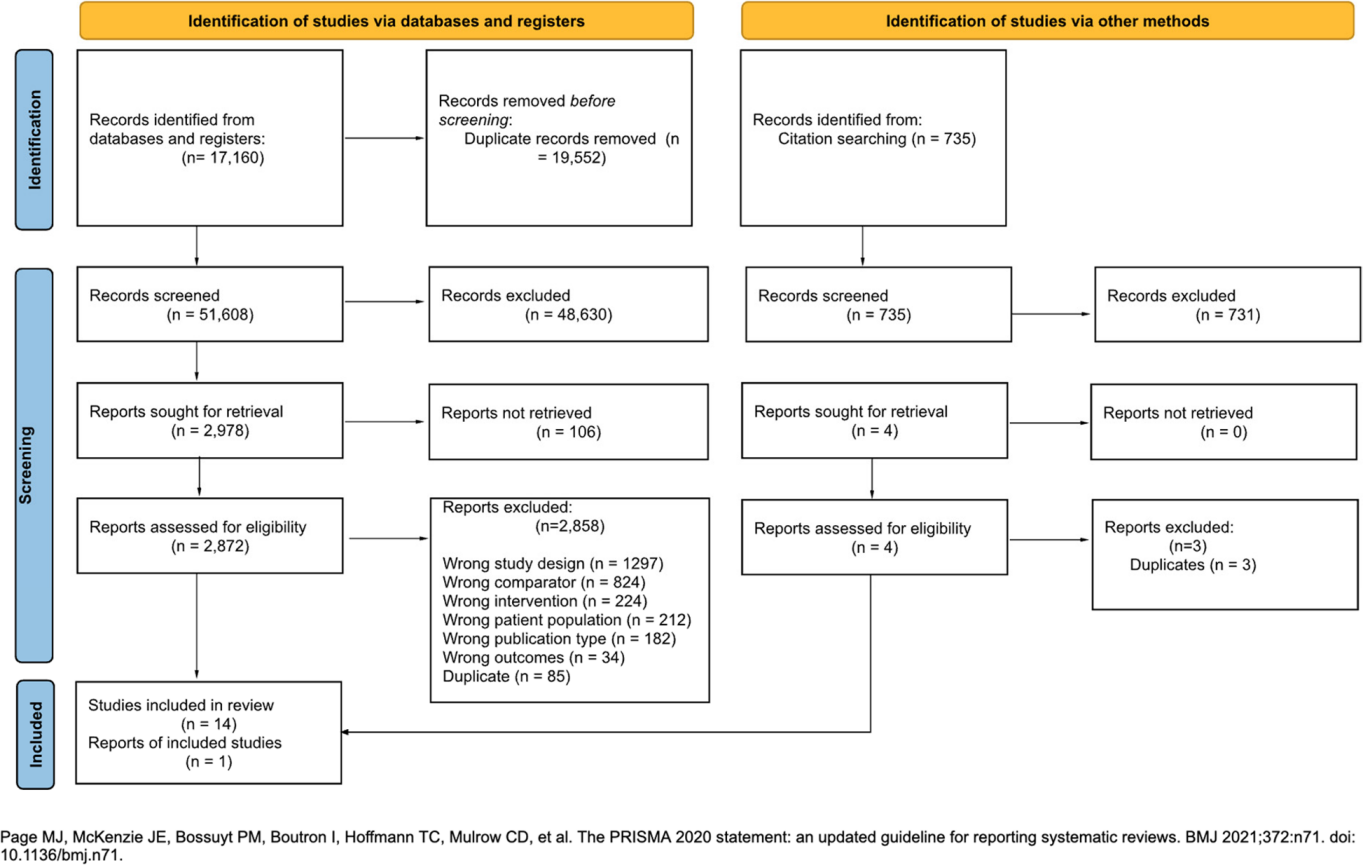
PRISMA 2020 flowchart

Our search identified 71,160 records. After removing duplicates, we assessed 51,608 references by title and abstract, excluding 48,630 references. We sought 2978 articles for full-text assessment, of which we included 14 studies [31–44]. Our citation search strategy identified one additional study that met the eligibility criteria [45]. Therefore, we finally included a total of 15 studies (*n* = 4329 patients). Figure 1 presents an overview of the selection process.

### Study characteristics

Table 1 summarises the general characteristics of the 15 included RCTs [31–45], all published between 1982 and 2021. Seven trials were multinational studies that included even more than 20 countries [34–38, 40, 43]. Most studies (10 out of 15) reached at least 1-year of follow-up. Private funding was involved in almost all studies (12 out of 15). No major differences in sex, age, and Eastern Collaborative Oncology Group (ECOG) performance status were observed across the included studies, except for three studies (the oldest) that included mostly patients with very poor performance status [31, 39, 43]. Some heterogeneity in inclusion criteria regarding cancer histology and location was identified, with studies in patients with adenocarcinoma predominating over squamous cell carcinoma. Only six studies enrolled exclusively patients with EC [31, 32, 39, 43–45].

**Table 1:** Characteristics of included studies (n= 15)

Author year (acronym)	Country(ies)	Centres (*n*)	Design (blind, phase)	Follow-up (months)	Funding	*N* included (intervention / control)	Population								Intervention	Previous treatment (lines)	Comparator	Outcomes of interest*
							Age	% Female	ESCC	ADC	EC	GEJ	GC	ECOG ≥ 2 (%)				
Alberts 1992 [31]	South Africa	1	NR	5	public and private	20 (10/10)	54.3	5.0	x		x			50.0	CT (5-FU, cisplatin) + RT + tube	NR	Observation + tube	OS, PFS, toxicity, QoL
Dutton 2014 (COG) [32]	UK	48	Double-blind phase III	12	public and private	450 (225/225)	64.8 (58, 70.7)	17.1	x	x	x	x		21.0	BIO/TT (gefitimib) + BSC	2nd or more	Placebo + BSC	OS, PFS, safety, HRQoL, DCR
Ford 2014 (COUGAR-02) [33]	UK	30	Open label phase III	18	private	168 (84/84)	65.5 (28, 84)	19.0		x	x	x	x	15.0	CT (docetaxel) + active control symptoms	2nd	BSC*	OS, PFS, HRQoL, toxicity, QoL
Fuchs 2014 (REGARD) [34]	multinational (29)	119	Double-blind placebo control phase III	12	private	355 (238/117)	60 (51, 71)	30.1				x	x	1.0	BIO/TT (ramucirumab) + BSC	2nd	Placebo + BSC	OS, PFS, PS, toxicity, symptoms related to the disease, QoL
Hall 2021 (GO2) [42]	UK	61	Open label phase III	12	public and private	45 (23/22)	78.6 (58, 89)	9.0		x	x	x	x	11.0	CT (oxaliplatin / capecitabine)	NR	BSC	OS, PFS,toxicity, symptoms related to the disease, QoL
Huang 2021 (ALTER1102) [45]	China	13	Double-blind placebo control phase II	18	private	165 (110/55)	61.5 (43, 76)	19.5	x		x			8.0	BIO/TT (anlotinib)	2nd or more	Placebo	OS, PFS, toxicity
Kang 2017 (ONO-4538–12, ATTRACTION-2) [35]	multinational (Japan, South Korea and Taiwan)	49	Double-blind placebo control phase III	18	private	493 (330/163)	61.5 (53, 69)	29.4		x		x	x	0.0	immunotherapy (nivolumab)	3rd or more	Placebo	OS, PFS, toxicity
Kang 2019 (ANGEL) [36]	multinational (13)	95	Double-blind placebo control phase III	36	private	460 (308/152)	60.0	23.3		x		x	x	0.0	BIO/TT (rivoceranib) + BSC	3rd or more	Placebo + BSC	OS, PFS, toxicity, QoL
Levard 1998 [43]	France	18	NR	14	NR	94 (48/46)	58.0	4.5	x		x			60.0	CT (5-FU, cisplatin)	NR	Observation	OS, toxicity
Li 2016 [41]	China	32	Double-blind placebo control phase III	30	private	267 (176/91)	58.0 (23, 71)	24.0		x		x	x	0.0	BIO/TT (apatinib)	3rd	Placebo	OS, PFS, toxicity, QoL
Nicolaou 1982 [44]	South Africa	1	NR	14	NR	24 (12/12)	57.0	0.0	x	x	x			0.0	CT (doxorubicin, cyclophosphamide)	NR	Observation + tube	OS, toxicity
Ohtsu 2013 (GRANITE-1) [37]	multinational (22)	137	Double-blind phase III	18	private	656 (439/217)	62.0 (20, 88)	26.4		x		x	x	0.1	BIO/TT (everolimus) + BSC	2nd or more	Placebo + BSC	OS, PFS, PS, toxicity, QoL
Pavlakis 2016 (INTEGRATE) [38]	multinational (4)	53	Double-blind placebo control phase II	6	public and private	147 (97/50)	60.0 (32, 85)	19.1		x		x	x	0.0	BIO/TT (regorafenib) + BSC	2nd or more	Placebo + BSC	OS, PFS, toxicity, symptoms related to disease, QoL
Schmid 1993 [39]	South Africa	1	NR	NR	NR	127 (40/46)	54.0	NR	x		x			68.6	CT (trimetrexate/ifosfamide + mesna/5-FU + leucovorin) + tube	NR	Observation + tube	OS, PS, toxicity, QoL
Shitara 2018 (TAGS) [40]	multinational (17)	110	Double-blind placebo control phase III	18	private	507 (337/170)	63.5 (56, 70)	28.0		x		x	x	0.0	CT (trifluridine / tipiracil) + BSC	3rd or more	Placebo + BSC	OS, PS, toxicity, symptoms related to disease, QoL

*5-FU, *5-fluorouracil;* ADC, *adenocarcinoma; *BIO/TT, *biological/targeted therapy; *BSC*, best supportive care; *Cis*, cisplatine; *CT*, chemotherapy; *DBPC*, double-blind placebo-controlled; *DCR*, disease control rate; *DTX*, docetaxel; *EC,* esophageal cancer; *ECOG*, Eastern Cooperative Oncology Group; *ESCC*, esophageal squamous cell carcinoma; *GC*, gastric cancer; *GEJ*, gastroesophageal junction; *HRQoL*, health-related quality of life; *irBORR*, immune-related best overall response rate; *irPFS*, immune-related progression-free survival; *OS*, overall survival; *ORR*, objective response rate; *PFS*, progression free survival; *PS*, performance status; *RT*, radiotherapy; *NR*, not reported; *QoL*, quality of life; *RCT*, randomised controlled trial. *Defined as active control symptoms

Regarding the anticancer drugs assessed, seven RCTs tested chemotherapy (5-FU, cisplatin, docetaxel, trimetrexate, trifluridine/tipiracil) [31, 33, 39, 40, 42–44], but only two of these reported the treatment line (2nd and 3rd) [33, 40]. Seven RCTs tested a second or third-line biological/targeted therapy regimen (anlotinib, apatinib, everolimus, gefitinib, ramucirumab, rivoceranib) [32, 34, 36–38, 41, 45]. Finally, only one study tested a third-line immunotherapy regimen (nivolumab) [35]. Most trials (9 out of 13) used placebo as the control group [32, 34–38, 40, 41, 45]. In the remaining cases, the comparison arm consisted of observation in four studies [31, 39, 43, 44], or supportive care exclusively in two studies [33, 34].

### Risk of *bias*

**Fig. 2:**
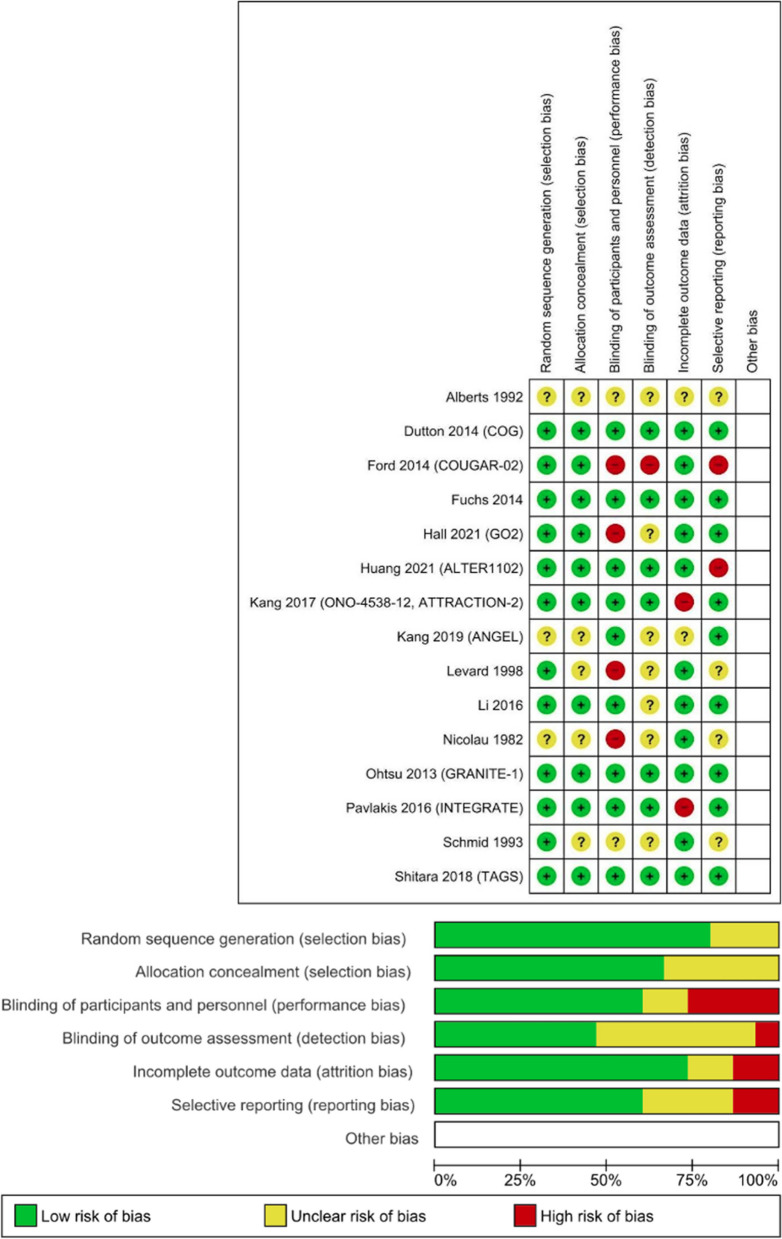
Risk of bias assessment (*n *= 15)

Four studies had an overall low RoB [32, 34, 40, 41], and five studies had an overall unclear RoB [31, 35–37, 39] (See Fig. 2 and Additional file 3 for an example assessment). The remaining six studies were considered as high risk of bias due to the lack of blinding of participants and personnel [33, 42–44], unblinded outcome assessment [33], incomplete outcome data [38], or selective reporting [33, 45].

### Efficacy outcomes

#### Overall survival (OS)

**Fig. 3:**
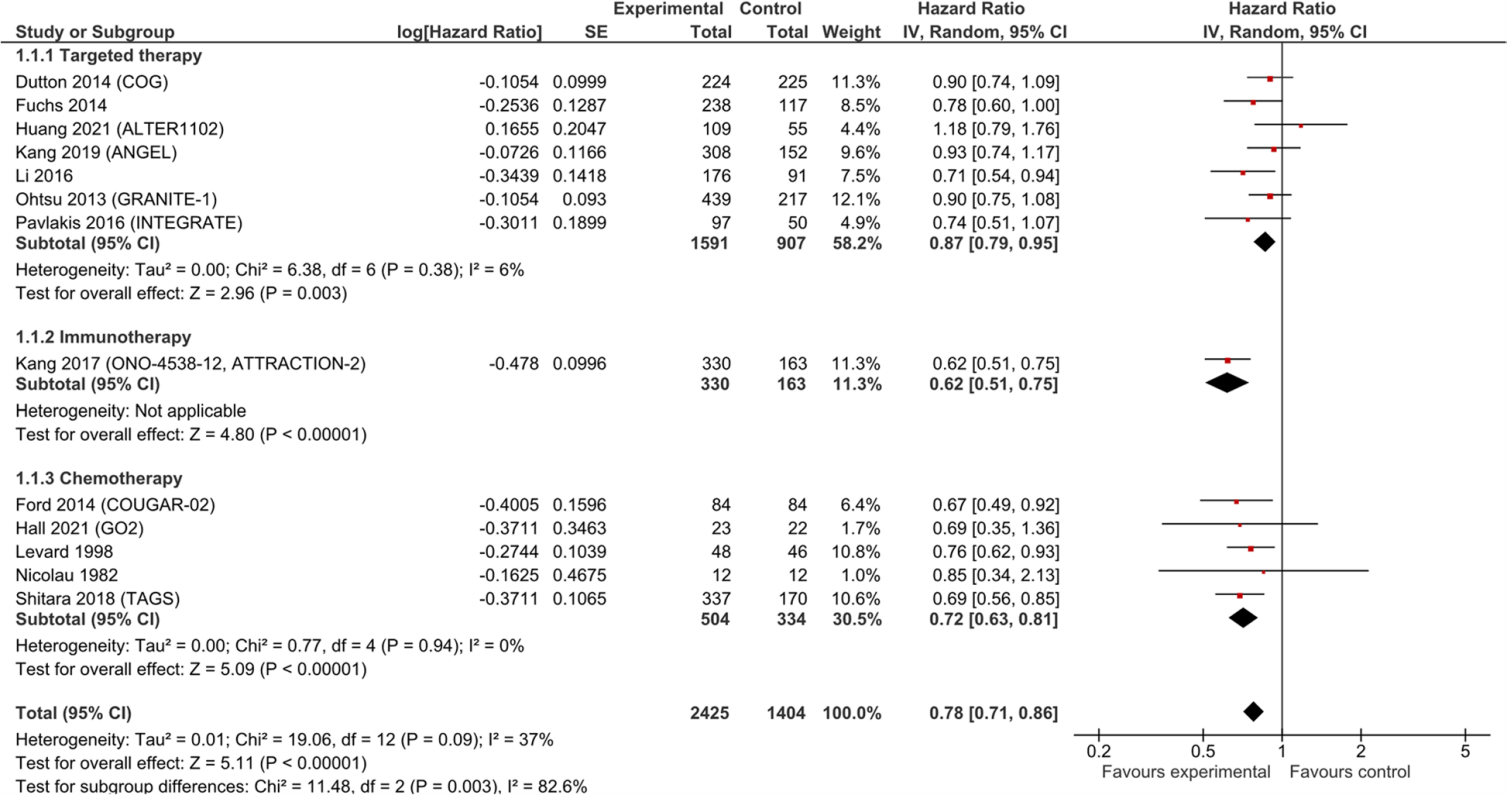
Overall survival in studies comparing anticancer drugs to supportive care for advanced esophageal cancer—by treatment type

Thirteen studies were included in the pooled analysis of OS (*n* = 3978). Alberts 1992 and Schmid 1993 [31, 39] did not report OS in sufficient detail to allow inclusion in the meta-analysis. Anticancer drugs, compared to supportive care, improved survival, with a pooled HR of 0.78 (95% CI 0.71, 0.86 *p* < 0.001; low certainty of evidence, Fig. 3), having a different effect depending on the type of drug. Immunotherapy (1 trial, 493 patients, HR 0.62; 95% CI 0.51, 0.75 *p* < 0.001; low certainty evidence) and chemotherapy (5 trials, 838 patients, HR 0.72; 95% CI 0.63, 0.81 *p* < 0.001; low certainty evidence) showed the largest effect, while biological/targeted therapy had the lowest (7 trials, 2498 patients, HR 0.87; 95% CI 0.79, 0.95 *p* = 0.003; low certainty evidence). The absolute benefits however were limited, with a mean OS gain of 0.83 months (95% CI 0.24, 1.42; low certainty evidence; Additional file 4). We conducted a sensitivity analysis of OS excluding Ford 2014 [33] (the study with high RoB in three domains), and found also a significant effect (HR 0.79; 95% CI 0.72, 0.87 *p* < 0.001; Additional file 4).

#### Progression-free survival (PFS) 

**Fig. 4:**
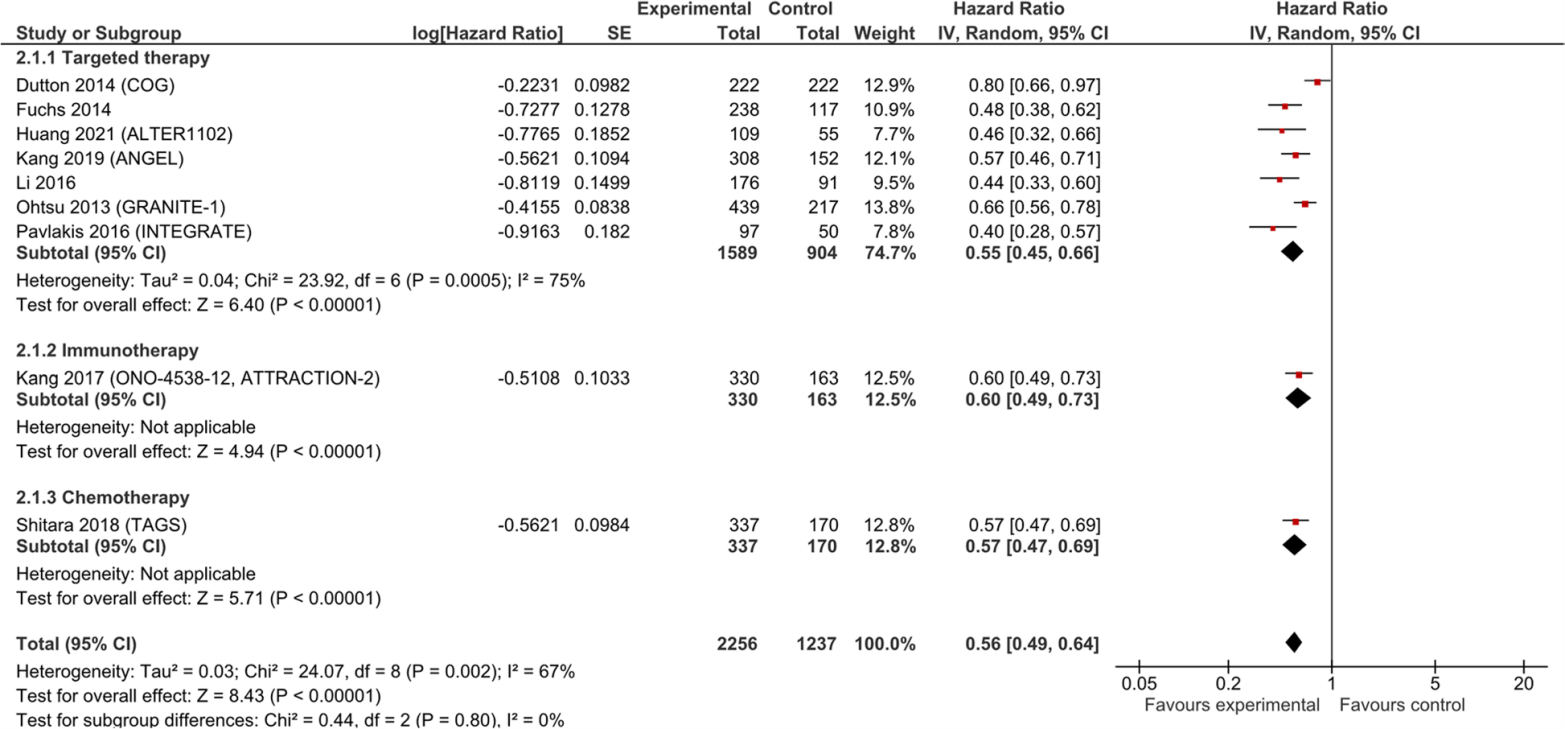
Progression-free survival in studies comparing anticancer drugs to supportive care for advanced esophageal cancer—by treatment type

Pooled analysis of PFS performed on data from nine RCTs (*n* = 3493) yielded a significant HR of 0.56 (95% CI 0.49, 0.64; low certainty evidence; Fig. 4). Alberts 1992, Ford 2014, Hall 2021, and Schmid 1993 did not report PFS in sufficient detail to be included in the meta-analysis. All treatment types showed benefit on PFS: immunotherapy (1 trial, 493 patients, HR 0.60; 95% CI 0.49, 0.73; low certainty evidence), chemotherapy (1 trial, 387 patients, HR 0.57; 95% CI 0.47, 0.69; low certainty evidence), and biological/targeted therapy (7 trials, 2493 patients, HR 0.55; 95% CI 0.45, 0.66; low certainty evidence). However, the absolute benefits were negligible, with a mean PFS gain of 0.68 months (95% CI 0.39, 0.97; low certainty evidence; Additional file 5). We performed sensitivity analyses excluding the studies that involved participants with both GEJ and gastric cancer patients [34–38, 40, 41]. When excluding these studies, PFS showed a non-significant effect (HR 0.62; 95% CI 0.36, 1.07; Additional file 5).

#### Performance status (PS)

**Fig. 5:**
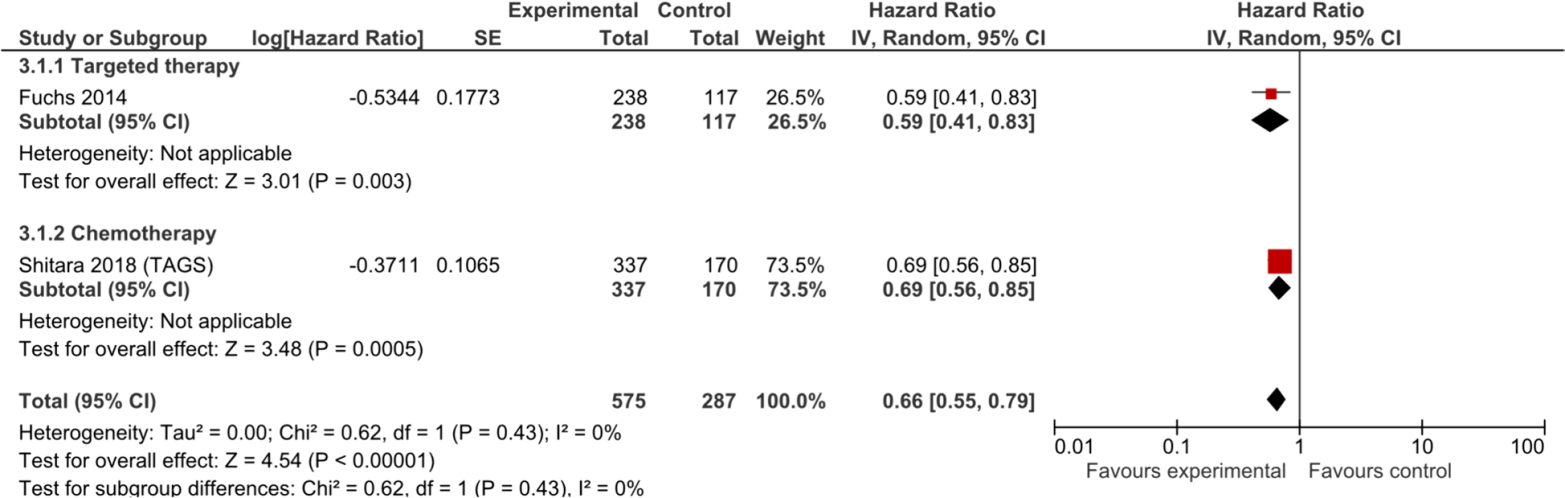
Performance status (time to deterioration) in studies comparing anticancer drugs to supportive care for advanced esophageal cancer—by treatment type

Time to deterioration of ECOG PS was improved by anticancer drugs, although with low certainty evidence. The pooled data from two RCTs comprising 862 patients yielded a significant HR of 0.66 (95% CI 0.55, 0.79; low certainty evidence; Fig. 5). Chemotherapy (1 trial, 507 patients, HR 0.69; 95% CI 0.56, 0.85; low certainty evidence) showed similar effect as biological/targeted therapy (1 trial, 355 patients, HR 0.59; 95% CI 0.41, 0.83; low certainty evidence).

### Safety outcomes

**Fig. 6:**
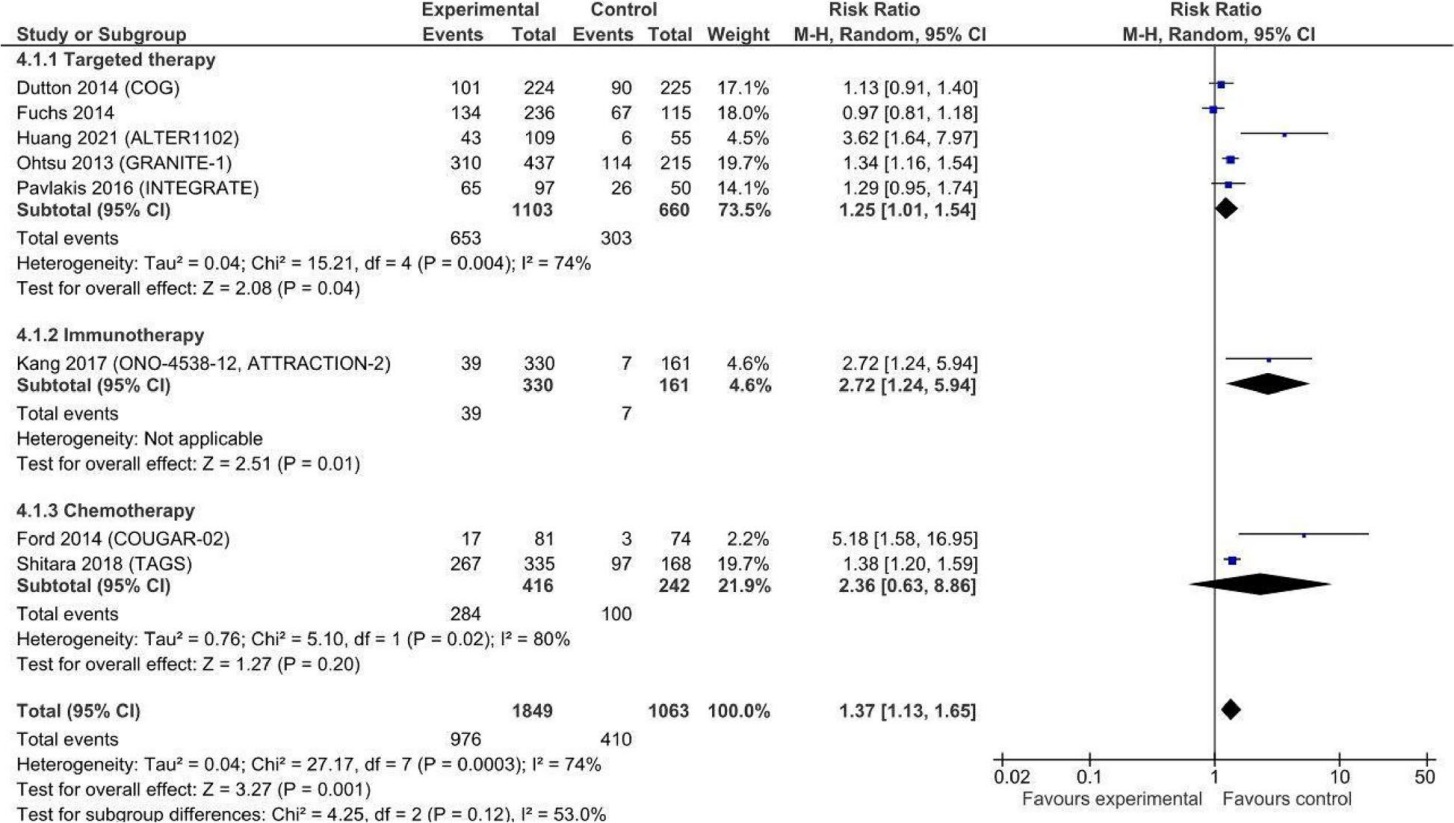
Toxicity in studies comparing anticancer drugs to supportive care for advanced esophageal cancer—by treatment

Overall grade 3–5 toxicity (8 trials, 2912 patients) was significantly increased with the addition of anticancer drugs, with a RR of 1.37 (95% CI 1.13, 1.65; very low certainty evidence; Fig. 6). The pooled toxicity rate was 52.8% (976/1849) in the experimental arm and 38.6% (410/1063) in the control arm. There was evidence of substantial heterogeneity (*I*^2^ = 74%, *p* < 0.03). Both immunotherapy (1 trial, 491 patients, RR 2.72; 95% CI 1.24, 5.94; low certainty evidence) and biological/targeted therapy (5 trials, 956 patients, RR 1.25; 95% CI 1.01, 1.54; very low certainty evidence) showed an increased risk of toxicity. Studies on chemotherapy also showed an increased risk of toxicity, but the meta-analysis did not show significance.

### Quality of life (QoL)

Nine of the identified trials reported QoL data [32–34, 36–38, 40–42] (Additional file 6) using validated QoL scales (EORTC QLQ-C30 –global and subscales, EORTC QLQ-STO22, EORTC QLQ-OG25, EQ-5D-3L). Due to heterogeneity in the report among the included trials, we describe this outcome narratively. Seven studies reported no significant differences between groups. Only one study reported significant improvement in QoL [33]—for docetaxel—in all pre-specified important domains, of which dysphagia was statistically significant (*p* = 0.02), and for several exploratory domains. Patients in the docetaxel group reported less general pain (*p* = 0.0008), abdominal pain (*p* = 0.01), nausea and vomiting (*p* = 0.02), and constipation (*p* = 0.02) than those in the control group, but similar global QoL (*p* = 0.53) [33]. Notably, one study [36] mentioned in their methods that QoL would be assessed, but the available conference abstract do not include information on this outcome.

### Certainty of the evidence

Full details of the analyses and their GRADE ratings can be found in the SoF tables (Additional file 7). The first comparison—chemotherapy versus supportive care—presents results for all outcomes. Comparison 2—immunotherapy versus supportive care—shows results for OS, PFS, and toxicity. The final comparison—biological/targeted therapy versus supportive care—presents data for the OS, PFS, toxicity, and QoL. The certainty of the evidence for all the outcomes was downgraded two levels to reflect the indirectness of the results, since RCTs included GEJ and gastric population among their included patients. We also downgraded a further level due to imprecision in some outcomes, so overall, we judged the evidence to be of low or very low certainty.

## Discussion

This SR identified, evaluated, and summarised the results of a total of 15 RCTs that assessed the efficacy and safety of anticancer drugs compared to supportive care for treating advanced esophageal cancer. However, interpreting and extracting meaningful insights from available studies was challenging, since most of them did not specifically focus on EC, lacked comprehensive details on treatment lines, failed to evaluate all relevant outcomes, and reported only negligible benefits. As a consequence, the certainty of the evidence is low or very low.

Having in mind those studies’ drawbacks, the results of our SR point out that anticancer drugs—chemotherapy, immunotherapy, and biological/targeted therapy—may exhibit a notable reduction in the risk of death by 21.0% and disease progression by 44.0%. However, it is important to note that this reduction in risk is accompanied by a 1.37 times increased risk of toxicity compared to supportive care. The analysis of the QoL outcome (which was not meta-analysed) showed little to no effect. Nevertheless, the observed benefit in terms of survival and disease control is modest, since absolute benefits on OS (0.8 months) and PFS (0.7 months) represent mean gains of less than a month. The certainty of the evidence was judged from low to very low across the different outcomes mainly due to the indirectness of results (RCTs included both GEJ and gastric population among their cases) and imprecision. Our SR shows a lack of information on other patient important outcomes beyond survival, toxicity, and QoL. Despite actively seeking that information, very few RCTs reported treatment effect on PS, and none reported hospital admissions or quality of end-of-life care. The observation that there were almost as many different agents used as there were trials highlights a significant challenge in comparing treatment outcomes across different agents. This diversity in treatment regimens makes direct comparisons between agents extremely difficult, underscoring the complexity of interpreting the results and drawing definitive conclusions regarding treatment efficacy.

Our study reveals the HR for the three broad treatment categories were very similar for PFS, but targeted therapy seems to have a higher HR than chemotherapy for OS. The consistent HRs for PFS underscore the efficacy of diverse treatment approaches in controlling tumour growth. Yet, the discrepancy in OS HRs prompts exploration into the distinct mechanisms of targeted therapy versus chemotherapy. We note limitations, including the variability in treatment responses and the low certainty of evidence. Further research with larger cohorts is essential to validate our findings and guide personalised treatment strategies for advanced patients. Our analysis also revealed a predominance of chemotherapy trials in EC and targeted therapy trials in gastric and GEJ cancer, reflecting the distinct therapeutic approaches based on tumour location. The impact of cancer location on treatment outcomes is evident from our findings. For chemotherapy, we observed a significant improvement in OS only in trials including cancers of the GEJ (two studies), with no significant benefit seen in pure EC (one study) or esophagus plus junction (one study). This suggests that the efficacy of chemotherapy may be influenced by the anatomical location of the tumour, highlighting the need for tailored treatment approaches based on tumour site. Similarly, our analysis of targeted therapy trials revealed no statistically significant OS improvement across different cancer locations, including esophageal (one study), junction (two studies), and esophagus plus junction (one study). These findings emphasise the complexity of treatment response in different tumour microenvironments and the challenges in achieving uniform efficacy across diverse cancer types. The observed high heterogeneity could stem from several factors. Firstly, the included studies lacked specificity and detailed information regarding treatment lines and evaluation of all relevant outcomes for advanced EC patients. Secondly, anticancer drugs encompass a broad spectrum of therapies, including chemotherapy, immunotherapy, and targeted therapy, each with distinct mechanisms of action, efficacy profiles, and toxicity profiles. This diversity in treatment modalities can result in variability in treatment responses across studies. Thirdly, most studies did not specifically focus on EC, which may have introduced variability in the results. Moreover, patients with advanced EC may exhibit considerable heterogeneity in disease characteristics, such as tumour histology, stage, comorbidities, demographic factors, and treatment histories.

There are several SRs published on the efficacy and safety of anticancer drugs for advanced EC [18, 46–48]. Nevertheless, not all of them included a comparison with only supportive care measures. The Cochrane systematic review by Janmmat et al. (2017) [48] evaluated the effects of palliative chemotherapy or palliative targeted therapy for esophageal or GEJ cancer. They found five studies comparing chemotherapy or targeted therapy plus best supportive care (BSC) versus BSC, using the following agents: cyclophosphamide plus doxorubicin (Nicolaou 1982), 5-FU plus cisplatin (Levard 1998), docetaxel (Ford 2014), ramucirumab (Fuchs 2014), and gefitinib (Dutton 2014). Two studies were first-line therapy regimens (Nicolaou 1982; Levard 1998), and the others were second-line regimens. The main difference between this review and ours consists in their inclusion of studies that used palliative chemotherapy as a comparator, which does not address the question of whether anticancer drugs are more effective than supportive care. Additionally, our review also assesses both immunotherapy and targeted therapy and includes more recent studies.

Although Akhlaghi et al. (2020) reported that receiving palliative chemotherapy was associated with reduced QoL in patients with advanced cancer at the end of life [49], drawing a conclusion regarding QoL towards the end of life could be biased, and both the Cochrane review by Janmaat et al. (2017) and an overview published by our research team (Santero 2021) lacked to detect that palliative chemotherapy and/or targeted therapy decrease QoL [18, 48]. This probably can be explained by the lack of sensitivity of the included trials to detect changes in most of the qualitative variables. However, all the studies agree that the risk of treatment-related grade 3 or higher toxicities was higher with chemotherapy or targeted therapy. Our SR revealed that only three RCTs have assessed QoL for the comparison of chemotherapy versus supportive care, with very uncertain findings about its effects, while six RCTs that compared biological/targeted therapies versus supportive care reveal that there may be no significant differences between groups. Also, for both comparisons, the certainty of evidence for toxicity was very low.

This study is one of the few SRs assessing the effect of anticancer drugs compared to supportive care for patients with advanced EC considering patient important outcomes apart from those related to survival and toxicity. We undertook a comprehensive search in five databases without any language or date restriction to minimise selection bias. The eligibility and data extraction processes were conducted by two independent reviewers to minimise errors. We assessed the RoB and certainty of the evidence for each outcome using an internationally recognised methodology (the GRADE approach [27]), providing a reasoned judgement for the confidence in each effect size to facilitate its use in decision-making. Furthermore, we carried out some subgroup analyses based on the type of therapy, which provides very relevant information that helps to choose one or another alternative.

Despite its strengths, this SR has potential limitations that should be acknowledged. First, as it has been made explicit before, the included studies exhibited severe limitations in terms of internal validity. Moreover, while most evidence is provided for adenocarcinoma, squamous cell carcinoma is much more frequent. We included GEJ adenocarcinoma among the eligibility criteria of our review because it was not always possible to extract the proposed primary endpoints for patients with EC alone. Additionally, most trials did not include frail or elderly patients, which can lead to inappropriate generalisation to them, since anticancer drugs are almost always investigated on less frail and younger patients [46]. Third, the inclusion of studies that did not separately report the effects of different lines and treatment strategies is another limitation that can compromise the certainty of the findings, emphasising the need for caution when interpreting the findings.

## Conclusions

In conclusion, our SR highlights the challenges of interpreting and deriving meaningful conclusions from studies that lack specificity for advanced EC, insufficiently detail the treatment lines examined, fail to evaluate all relevant outcomes, or demonstrate only minimal treatment benefits. As a result, the certainty of the conclusions drawn is low or very low, and therefore, careful consideration of the potential benefits and risks of anticancer drugs for treating people with advanced EC is essential. Our SR identifies essential gaps in the treatment of advanced EC patients, underscoring the importance of designing more and better RCTs that allow a valid comparison between any systemic therapy and supportive cares in relation to their benefits and harms.

### Supplementary Information

Additional file 1: Eligibility criteria.

Additional file 2: Search strategy.

Additional file 3: RoB assessment example.

Additional file 4: Overall survival (OS) outcome: Sensitivity analysis and meta-analyses (continuous, 6, 12, and 18 months).

Additional file 5: Progression-free survival (PFS) outcome: Sensitivity analysis and meta-analyses (continuous, 6, 12, and 18 months).

Additional file 6: Quality of life (QoL) in studies comparing anticancer drugs to supportive care for advanced esophageal cancer.

Additional file 7: GRADE assessment and Summary of Findings (SoF) tables.

## Data Availability

Pérez JB, Salazar J, Santero M, Requeijo C, Grijalva GR, Acosta-Dighero R, et al. Efficacy of systemic oncological treatments in patients with advanced, non-intestinal digestive cancer at high risk of dying in the middle and short term: evidence synthesis (ASTAC-study). Open Science Framework; 2022. 10.17605/OSF.IO/7CHX6

## Abbreviations

AEs: Adverse events
BSC: Best supportive care
CENTRAL: Cochrane Central Register of Controlled Trials
CI: Confidence interval
CTCAE: Common Terminology Criteria for Adverse Events
EC: Esophageal cancer
ECOG: Eastern Collaborative Oncology Group
EMA: European Medicine Agency
FDA: Food and Drug Administration
GEJ: Gastroesophageal junction
GRADE: Grading of Recommendations Assessment, Development, and Evaluation
HR: Hazard ratio
MD: Mean difference
OS: Overall survival
OSF: Open Science Framework
PFS: Progression-free survival
PRISMA: Preferred Reporting Items for Systematic Reviews and Meta-Analyses
PROSPERO: International prospective register of systematic reviews
PS: Performance status
QoL: Quality of life
RCTs: Randomised controlled trials
RR: Risk ratio
SoF: Summary of Findings
SR: Systematic review
