# Supplementary material for: Utilising systematic reviews to assess potential overtreatment and claim for better evidence-based research: an analysis of anticancer drugs versus supportive care in advanced esophageal cancer
Source: Syst Rev. 2024 Jul 18;13:186. doi: 10.1186/s13643-024-02594-1 (PMC11256491; doi:10.1186/s13643-024-02594-1)
Supplement: Supplementary file 1 — Additional file 1: Eligibility criteria. [file 13643_2024_2594_MOESM1_ESM.pdf]

Summarised eligibility criteria

PICO	Inclusion criteria	Exclusion criteria
Patients	adults with an advanced primary oesophageal cancer (including gastroesophageal junction) as defined by study authors or according to the staging IIIb, IIIc, and IV	neuroendocrine, stromal or lymphatic neoplasms
Interventions	any chemotherapy, either monotherapy or in combination, or biological/targeted therapy, or immunotherapy, whether individual or combined, with or without supportive care	surgery or radiotherapy as sole interventions. Adjuvant or neoadjuvant chemotherapy
Comparisons	any supportive treatment, administered with the purpose of symptomatic or palliative control, including BSC, placebo, or non-specified control intervention	non-palliative treatments
Outcomes	survival; quality of life; functional status; toxicity; progression-free survival; symptoms related to the disease; admissions to hospital or long-term centre, or emergency consultations; quality of end-of-life care	
Type of studies	randomised clinical trials	quasi-experimental studies, observational studies, reviews, protocols
