# Supplementary material for: Utilising systematic reviews to assess potential overtreatment and claim for better evidence-based research: an analysis of anticancer drugs versus supportive care in advanced esophageal cancer
Source: Syst Rev. 2024 Jul 18;13:186. doi: 10.1186/s13643-024-02594-1 (PMC11256491; doi:10.1186/s13643-024-02594-1)
Supplement: Supplementary file 2 — Additional file 2: Search strategy. [file 13643_2024_2594_MOESM2_ESM.pdf]

Search strategy

MEDLINE/PubMed<br>Date of last search: December 2, 2019	
#1	("Gastrointestinal Neoplasms"[Mesh:NoExp] OR "Esophageal Neoplasms"[Mesh] OR "Stomach Neoplasms"[Mesh] OR "Liver Neoplasms"[Mesh] OR "Biliary Tract Neoplasms"[Mesh] OR "Pancreatic Neoplasms"[Mesh])
#2	((esophag*[Title] OR oesophag*[Title] OR stomach*[Title] OR gastric*[Title] OR gastroesophag*[Title] OR liver*[Title] OR hepatic*[Title] OR hepatocel*[Title] OR biliary tract*[Title] OR bile duct*[Title] OR gallbladder*[Title] OR gall bladder*[Title] OR pancreas*[Title] OR pancreatic*[Title] OR gastrointestinal*[Title]) AND (cancer*[Title] OR carcinom*[Title] OR neoplasm*[Title] OR tumor*[Title] OR tumour*[Title] OR malignan*[Title] OR adenocar*[Title] OR oncolog*[Title]))
#3	(#1 OR #2)
#4	("Palliative Care"[Mesh] OR "Terminal Care"[Mesh] OR "Neoplasm Metastasis"[Mesh])
#5	(palliative*[Title/Abstract] OR end of life*[Title/Abstract] OR end of live*[Title/Abstract] OR terminal*[Title/Abstract] OR metasta*[Title/Abstract] OR BSC[Title/Abstract] OR supportive care*[Title/Abstract] OR advanced*[Title/Abstract] OR unresect*[Title/Abstract] OR irresect*[Title/Abstract] OR nonresect*[Title/Abstract] OR non resect*[Title/Abstract] OR inopera*[Title/Abstract] OR unopera*[Title/Abstract] OR nonopera*[Title/Abstract] OR non opera*[Title/Abstract] OR non-opera*[Title/Abstract] OR stage IV[Title/Abstract])
#6	(#4 OR #5)
#7	(#3 AND #6)
#8	("Antineoplastic Protocols"[Mesh] OR "Chemoradiotherapy"[Mesh] OR "Induction Chemotherapy"[Mesh] OR "Maintenance Chemotherapy"[Mesh] OR "Consolidation Chemotherapy"[Mesh])
#9	(antineoplastic*[Title] OR antineoplasic*[Title] OR chemotherap*[Title] OR chemoradiotherap*[Title] OR radiochemotherap*[Title] OR carboplatin*[Title] OR cisplatin*[Title] OR oxaliplatin*[Title] OR platin*[Title] OR fluorouracil*[Title] OR 5-FU[Title] OR capecitabine*[Title] OR docetaxel*[Title] OR taxotere[Title] OR epirucibin*[Title] OR irinotecan*[Title] OR onivyde[Title] OR paclitaxel*[Title] OR abraxane[Title] OR trifluridine*[Title] OR tipiracil*[Title] OR lonsurf[Title] OR gemcitabine*[Title] OR gemzar[Title] OR mitomycin*[Title])
#10	(#8 OR #9)
#11	("Molecular Targeted Therapy"[Mesh] OR "Antibodies, Monoclonal"[Mesh] OR "Cancer Vaccines"[Mesh])
#12	(Target*[Title] OR antibod*[Title] OR immunotherap*[Title] OR vaccine[Title] OR vaccines[Title] OR vaccination[Title] OR tyrosine kinase inhibit*[Title] OR trastuzumab[Title] OR herceptin[Title] OR bevacizumab[Title] OR rilotumumab[Title] OR onartuzumab[Title] OR ramucirumab[Title] OR cyramza[Title] OR cetuximab[Title] OR panitumumab[Title] OR nimotuzumab[Title] OR claudiximab[Title] OR

	apatinib[Title] OR lapatinib[Title] OR regorafenib[Title] OR stivarga[Title] OR everolimus[Title] OR nivolumab[Title] OR opdivo[Title] OR pembrolizumab[Title] OR keytruda[Title] OR avelumab[Title] OR durvalumab[Title] OR ipilimumab[Title] OR checkpoint inhibit*[Title] OR cabozantinib[Title] OR cabometyx[Title] OR lenvatinib[Title] OR lenvima[Title] OR sorafenib[Title] OR nexavar[Title] OR sunitinib[Title] OR sutent[Title] OR erlotinib[Title] OR tarceva[Title] OR doxorubicin[Title])
#13	(#11 OR #12)
#14	(#10 OR #13)
#15	(#7 AND #14)
#16	(animals [mh] NOT humans [mh])
#17	(#15 NOT #16)

EMBASE/Ovid<br>Date of last search: December 2, 2019	
#1	*digestive system cancer/
#2	exp *esophagus cancer/
#3	exp *stomach cancer/
#4	exp *hepatobiliary system cancer/
#5	exp *pancreas cancer/
#6	((esophag* or oesophag* or stomach* or gastric* or gastroesophag* or liver* or hepatic* or hepatocel* or biliary tract* or bile duct* or gallbladder* or gall bladder* or pancreas* or pancreatic* or gastrointestinal*) and (cancer* or carcinom* or neoplasm* or tumor* or tumour* or malignan* or adenocar* or oncolog*)).ti.
#7	1 or 2 or 3 or 4 or 5 or 6
#8	exp *cancer palliative therapy/
#9	exp *terminal care/
#10	exp *metastasis/
#11	(palliative* or end NEXT of NEXT life* or end-of-life or terminal NEXT care* or metasta* or BSC or supportive NEXT care* or advanced* or unresect* or irresect* or nonresect* or non NEXT resect* or inopera* or unopera* or nonopera* or non NEXT opera* or non-opera* or stage IV).ti.
#12	8 or 9 or 10 or 11
#13	exp *cancer chemotherapy/

#14	(antineoplastic* or antineoplasic* or chemotherap* or chemoradiotherap* or radiochemotherap* or carboplatin* or cisplatin* or oxaliplatin* or platin* or fluorouracil* or 5-fu or capecitabine or docetaxel or taxotere or epirucibin or irinotecan or onivyde or paclitaxel or abraxane or trifluridine or tipiracil or lonsurf or gemcitabine or gemzar or mitomycin).ti.
#15	exp *antineoplastic agent/
#16	exp *molecularly targeted therapy/
#17	exp *cancer immunotherapy/
#18	(target* or antibod* or immunotherap* or vaccine or vaccines or vaccination or tyrosine kinase inhibit* or trastuzumab or herceptin or bevacizumab or rilotumumab or onartuzumab or ramucirumab or cyramza or cetuximab or panitumumab or nimotuzumab or claudiximab or apatinib or lapatinib or regorafenib or stivarga or everolimus or nivolumab or opdivo or pembrolizumab or keytruda or avelumab or durvalumab or ipilimumab or checkpoint inhibit* or cabozantinib or cabometyx or lenvatinib or lenvima or sorafenib or nexavar or sunitinib or sutent or erlotinib or tarceva or doxorubicin).ti.
#19	13 or 14 or 15 or 16 or 17 or 18
#20	7 and 12 and 19

Cochrane Library<br>Date of last search: December 3, 2019	
#1	MeSH descriptor: [Gastrointestinal Neoplasms] this term only
#2	MeSH descriptor: [Esophageal Neoplasms] explode all trees
#3	MeSH descriptor: [Stomach Neoplasms] explode all trees
#4	MeSH descriptor: [Liver Neoplasms] explode all trees
#5	MeSH descriptor: [Biliary Tract Neoplasms] explode all trees
#6	MeSH descriptor: [Pancreatic Neoplasms] explode all trees
#7	(((esophag* OR oesophag* OR stomach* OR gastric* OR gastroesophag* OR liver* OR hepatic* OR hepatocel* OR biliary tract* OR bile duct* OR gallbladder* OR gall bladder* OR pancreas* OR pancreatic* OR gastrointestinal*) AND (cancer* OR carcinom* OR neoplasm* OR tumor* OR tumour* OR malignan* OR adenocar* OR oncolog*))):ti (Word variations have been searched)
#8	#1 OR #2 OR #3 OR #4 OR #5 OR #6 OR #7

#9	MeSH descriptor: [Palliative Care] explode all trees
#10	MeSH descriptor: [Terminal Care] explode all trees
#11	MeSH descriptor: [Neoplasm Metastasis] explode all trees
#12	((palliative* OR (end of life*) OR (end of live*) OR terminal* OR metasta* OR BSC OR (supportive NEXT care*) OR advanced* OR unresect* OR irresect* OR nonresect* OR (non NEXT resect*) OR inopera* OR unopera* OR nonopera* OR (non NEXT opera*) OR non-opera* OR (stage IV))):ti (Word variations have been searched)
#13	#9 OR #10 OR #11 OR #12
#14	#8 AND #13
#15	MeSH descriptor: [Chemoradiotherapy] explode all trees
#16	MeSH descriptor: [Consolidation Chemotherapy] explode all trees
#17	MeSH descriptor: [Induction Chemotherapy] explode all trees
#18	MeSH descriptor: [Maintenance Chemotherapy] explode all trees
#19	MeSH descriptor: [Antineoplastic Protocols] explode all trees
#20	((antineoplastic* OR antineoplasic* OR chemotherap* OR chemoradiotherap* OR radiochemotherap* OR carboplatin* OR cisplatin* OR oxaliplatin* OR platin* OR fluorouracil* OR capecitabine* OR docetaxel* OR taxotere OR epirucibin* OR irinotecan* OR onivyde OR paclitaxel* OR abraxane OR trifluridine* OR tipiracil* OR lonsurf OR gemcitabine* OR gemzar OR mitomycin*)):ti (Word variations have been searched)
#21	#15 OR #16 OR #17 OR #18 OR #19 OR #20
#22	MeSH descriptor: [Molecular Targeted Therapy] explode all trees
#23	MeSH descriptor: [Antineoplastic Agents] explode all trees
#24	MeSH descriptor: [Cancer Vaccines] explode all trees
#25	((Target* OR antibod* OR immunotherap* OR vaccine OR vaccines OR vaccination OR (tyrosine kinase inhibit*) OR trastuzumab OR herceptin OR bevacizumab OR rilotumumab OR onartuzumab OR ramucirumab OR cyramza OR cetuximab OR panitumumab OR nimotuzumab OR claudiximab OR apatinib OR lapatinib OR regorafenib OR stivarga OR everolimus OR nivolumab OR opdivo OR pembrolizumab

	OR keytruda OR avelumab OR durvalumab OR ipilimumab OR (checkpoint inhibit*) OR cabozantinib OR cabometyx OR lenvatinib OR lenvima OR sorafenib OR nexavar OR sunitinib OR sutent OR erlotinib OR tarceva OR doxorubicin)):ti (Word variations have been searched)
#26	#22 OR #23 OR #24 OR #25
#27	#21 OR #26
#28	#14 AND #27
#29	#28 in Cochrane Reviews, Cochrane Protocols, Trials

Epistemonikos<br>Date of last search: December 2, 2019
(title:(title:((esophag* OR oesophag* OR stomach* OR gastric* OR gastroesophag* OR liver* OR hepatic* OR hepatocel* OR biliary* OR bile* OR gallbladder* OR pancreas* OR pancreatic* OR gastrointestinal*) AND (cancer* OR carcinom* OR neoplasm* OR tumor* OR tumour* OR malignan* OR adenocar* OR oncolog*)) AND title:(palliative* OR terminal* OR metasta* OR supportive* OR advanced* OR unresect* OR irresect* OR nonresect* OR inopera* OR unopera* OR nonopera*) AND title:(antineoplastic* OR antineoplasic* OR chemotherap* OR chemoradiotherap* OR radiochemotherap* OR carboplatin* OR cisplatin* OR oxaliplatin* OR platin* OR fluorouracil* OR capecitabine OR docetaxel OR taxotere OR epirucibin OR irinotecan OR onivyde OR paclitaxel OR abraxane OR trifluridine OR tipiracil OR lonsurf OR gemcitabine OR gemzar OR mitomycin OR target* OR antibod* OR immunotherap* OR vaccine OR vaccines OR vaccination OR trastuzumab OR herceptin OR bevacizumab OR rilotumumab OR onartuzumab OR ramucirumab OR cyramza OR cetuximab OR panitumumab OR nimotuzumab OR claudiximab OR apatinib OR lapatinib OR regorafenib OR stivarga OR everolimus OR nivolumab OR opdivo OR pembrolizumab OR keytruda OR avelumab OR durvalumab OR ipilimumab OR cabozantinib OR cabometyx OR lenvatinib OR lenvima OR sorafenib OR nexavar OR sunitinib OR sutent OR erlotinib OR tarceva OR doxorubicin)) OR abstract:(title:((esophag* OR oesophag* OR stomach* OR gastric* OR gastroesophag* OR liver* OR hepatic* OR hepatocel* OR biliary* OR bile* OR gallbladder* OR pancreas* OR pancreatic* OR gastrointestinal*) AND (cancer* OR carcinom* OR neoplasm* OR tumor* OR tumour* OR malignan* OR adenocar* OR oncolog*)) AND title:(palliative* OR terminal* OR metasta* OR supportive* OR advanced* OR unresect* OR irresect* OR nonresect* OR inopera* OR unopera* OR nonopera*) AND title:(antineoplastic* OR antineoplasic* OR chemotherap* OR chemoradiotherap* OR radiochemotherap* OR carboplatin* OR cisplatin* OR oxaliplatin* OR platin* OR fluorouracil* OR capecitabine OR docetaxel OR taxotere OR epirucibin OR irinotecan OR onivyde OR paclitaxel OR abraxane OR trifluridine OR tipiracil OR lonsurf OR gemcitabine OR gemzar OR mitomycin OR target* OR antibod* OR immunotherap* OR vaccine OR vaccines OR vaccination OR trastuzumab OR herceptin OR bevacizumab OR rilotumumab OR onartuzumab OR ramucirumab OR cyramza OR cetuximab OR panitumumab OR nimotuzumab OR claudiximab OR apatinib OR lapatinib OR regorafenib OR stivarga OR everolimus OR nivolumab OR opdivo OR pembrolizumab OR keytruda OR avelumab

OR durvalumab OR ipilimumab OR cabozantinib OR cabometyx OR lenvatinib OR lenvima OR sorafenib OR nexavar OR sunitinib OR sutent OR erlotinib OR tarceva OR doxorubicin)))

clinicaltrials.gov<br>Date of last search: December 2, 2019
(advanced OR unresectable OR nonresectable OR unoperable OR inoperable OR stage IV) | (esophageal OR gastric OR stomach OR liver OR hepatocellular OR gallbladder OR bile duct OR pancreas OR pancreatic) AND (cancer OR neoplasm) | (chemotherapy OR targeted therapy OR target therapy OR tyrosine kinase inhibitor OR immunotherapy OR vaccine) AND (supportive care OR bsc OR placebo)

PROSPERO<br>Date of last search: December 7, 2019
(esophag* OR oesophag* OR stomach* OR gastric* OR gastroesophag* OR liver* OR hepatic* OR hepatocel* OR biliary tract* OR bile duct* OR gallbladder* OR gall bladder* OR pancreas* OR pancreatic* OR gastrointestinal*) AND (cancer* OR carcinom* OR neoplasm* OR tumor* OR tumour* OR malignan* OR adenocar* OR oncolog*) AND (palliative* OR end of life* OR end-of-life OR terminal care* OR metasta* OR BSC OR supportive care* OR advanced* OR unresect* OR irresect* OR nonresect* OR non resect* OR inopera* OR unopera* OR nonopera* OR non opera* OR non-opera* OR stage IV) AND (antineoplastic* OR antineoplasic* OR chemotherap* OR chemoradiotherap* OR radiochemotherap* OR carboplatin* OR cisplatin* OR fluorouracil* OR 5 FU OR capecitabine* OR docetaxel* OR epirucibin* OR irinotecan* OR oxaliplatin* OR paclitaxel* OR trifluridine* OR tipiracil* OR Target* OR antibod* OR immunotherap* OR vaccine OR vaccines OR vaccination OR HER2 OR HER-2 OR egfr OR VEGF* OR HGF OR MET OR claudin* OR MMP-9 OR tyrosine kinase inhibit* OR trastuzumab OR bevacizumab OR rilotumumab OR onartuzumab OR ramucirumab OR cetuximab OR panitumumab OR nimotuzumab OR claudiximab OR apatinib OR lapatinib OR regorafenib OR everolimus OR nivolumab OR pembrolizumab OR avelumab OR durvalumab OR ipilimumab OR checkpoint inhibit*)<br>Health area of review: Cancer, Care of the elderly, Palliative care<br>Restrict to specific fields Title [TI]

Updated search strategy

MEDLINE/PubMed<br>Date of last search: May 2022	
#1	("Esophageal Neoplasms"[Mesh] OR "Stomach Neoplasms"[Mesh])
#2	((esophag*[Title] OR oesophag*[Title] OR stomach*[Title] OR gastric*[Title] OR gastroesophag*[Title] OR gastrointestinal*[Title]) AND (cancer*[Title] OR carcinom*[Title] OR neoplasm*[Title] OR tumor*[Title] OR tumour*[Title] OR malignan*[Title] OR adenocar*[Title] OR oncolog*[Title]))

#3	(#1 OR #2)
#4	("Palliative Care"[Mesh] OR "Terminal Care"[Mesh] OR "Neoplasm Metastasis"[Mesh])
#5	(palliative*[Title/Abstract] OR end of life*[Title/Abstract] OR end of live*[Title/Abstract] OR terminal*[Title/Abstract] OR metasta*[Title/Abstract] OR BSC[Title/Abstract] OR supportive care*[Title/Abstract] OR advanced*[Title/Abstract] OR unresect*[Title/Abstract] OR irresect*[Title/Abstract] OR nonresect*[Title/Abstract] OR non resect*[Title/Abstract] OR inopera*[Title/Abstract] OR unopera*[Title/Abstract] OR nonopera*[Title/Abstract] OR non opera*[Title/Abstract] OR non-opera*[Title/Abstract] OR stage IV[Title/Abstract])
#6	(#4 OR #5)
#7	(#3 AND #6)
#8	("Antineoplastic Protocols"[Mesh] OR "Chemoradiotherapy"[Mesh] OR "Induction Chemotherapy"[Mesh] OR "Maintenance Chemotherapy"[Mesh] OR "Consolidation Chemotherapy"[Mesh])
#9	(antineoplastic*[Title] OR antineoplasic*[Title] OR chemotherap*[Title] OR chemoradiotherap*[Title] OR radiochemotherap*[Title] OR carboplatin*[Title] OR cisplatin*[Title] OR oxaliplatin*[Title] OR platin*[Title] OR fluorouracil*[Title] OR 5-FU[Title] OR capecitabine*[Title] OR docetaxel*[Title] OR taxotere[Title] OR epirubicin*[Title] OR irinotecan*[Title] OR onivyde[Title] OR paclitaxel*[Title] OR abraxane[Title] OR trifluridine*[Title] OR tipiracil*[Title] OR lonsurf[Title] OR gemcitabine*[Title] OR gemzar[Title] OR mitomycin*[Title])
#10	(#8 OR #9)
#11	("Molecular Targeted Therapy"[Mesh] OR "Antibodies, Monoclonal"[Mesh] OR "Cancer Vaccines"[Mesh])
#12	(Target*[Title] OR antibod*[Title] OR immunotherap*[Title] OR vaccine[Title] OR vaccines[Title] OR vaccination[Title] OR tyrosine kinase inhibit*[Title] OR trastuzumab[Title] OR herceptin[Title] OR bevacizumab[Title] OR rilotumumab[Title] OR onartuzumab[Title] OR ramucirumab[Title] OR cyramza[Title] OR cetuximab[Title] OR panitumumab[Title] OR nimotuzumab[Title] OR apatinib[Title] OR lapatinib[Title] OR regorafenib[Title] OR stivarga[Title] OR everolimus[Title] OR nivolumab[Title] OR opdivo[Title] OR pembrolizumab[Title] OR keytruda[Title] OR avelumab[Title] OR durvalumab[Title] OR ipilimumab[Title] OR checkpoint inhibit*[Title] OR cabozantinib[Title] OR cabometyx[Title] OR lenvatinib[Title] OR lenvima[Title] OR sorafenib[Title] OR nexavar[Title] OR sunitinib[Title] OR sutent[Title] OR erlotinib[Title] OR tarceva[Title] OR doxorubicin[Title])
#13	(#11 OR #12)
#14	(#10 OR #13)
#15	(#7 AND #14)
#16	(animals [mh] NOT humans [mh])
#17	(#15 NOT #16)

#18	randomized controlled trial[pt] OR controlled clinical trial[pt] OR randomized[tiab] OR placebo[tiab] OR clinical trials as topic[mesh:noexp] OR randomly[tiab] OR trial [ti]
#19	(#17 AND #18)
#20	2019/12/01:2022/05/06[pdat]
#21	(#19 AND #20)

Cochrane Central Register of Controlled Trials<br>Date of last search: May 2022	
#1	MeSH descriptor: [Gastrointestinal Neoplasms] explode all trees
#2	MeSH descriptor: [Esophageal Neoplasms] explode all trees
#3	MeSH descriptor: [Stomach Neoplasms] explode all trees
#4	(((esophag* OR oesophag* OR stomach* OR gastric* OR gastroesophag* OR gastrointestinal*) AND (cancer* OR carcinom* OR neoplasm* OR tumor* OR tumour* OR malignan* OR adenocar* OR oncolog*))):ti
#5	#1 OR #2 OR #3 OR #4
#6	MeSH descriptor: [Palliative Care] explode all trees
#7	MeSH descriptor: [Terminal Care] explode all trees
#8	MeSH descriptor: [Neoplasm Metastasis] explode all trees
#9	(palliative* OR “end of life” OR “end of live” OR terminal* OR metasta* OR BSC OR “supportive care” OR advanced* OR unresect* OR irresect* OR nonresec* OR (non NEXT resec*) OR inopera* OR unopera* OR nonopera* OR (non NEXT opera*) OR (stage NEXT IV)):ti,ab
#10	#6 OR #7 OR #8 OR #9
#11	#5 AND #10
#12	MeSH descriptor: [Antineoplastic Protocols] explode all trees
#13	MeSH descriptor: [Chemoradiotherapy] explode all trees
#14	MeSH descriptor: [Induction Chemotherapy] explode all trees
#15	MeSH descriptor: [Maintenance Chemotherapy] explode all trees
#16	MeSH descriptor: [Consolidation Chemotherapy] explode all trees
#17	(antineoplastic* OR antineoplasic* OR chemotherap* OR chemoradiotherap* OR radiochemotherap* OR carboplatin* OR cisplatin* OR oxaliplatin* OR platin* OR

	fluorouracil* OR (5 NEXT FU) OR capecitabine* OR docetaxel* OR taxotere OR epirubicin* OR irinotecan* OR onivyde OR paclitaxel* OR abraxane OR trifluridine* OR tipiracil* OR lonsurf OR gemcitabine* OR gemzar OR mitomycin*):ti
#18	#12 OR #13 OR #14 OR #15 OR #16 OR #17
#19	MeSH descriptor: [Molecular Targeted Therapy] explode all trees
#20	MeSH descriptor: [Antibodies, Monoclonal] explode all trees
#21	MeSH descriptor: [Cancer Vaccines] explode all trees
#22	(target* OR antibod* OR immunotherap* OR vaccine OR vaccines OR vaccination OR (tyrosine NEXT kinase NEXT inhibit*) OR trastuzumab OR herceptin OR bevacizumab OR rilotumumab OR onartuzumab OR ramucirumab OR cyramza OR cetuximab OR panitumumab OR nimotuzumab OR claudiximab OR apatinib OR lapatinib OR regorafenib OR stivarga OR everolimus OR nivolumab OR opdivo OR pembrolizumab OR keytruda OR avelumab OR durvalumab OR ipilimumab OR (checkpoint NEXT inhibit*) OR (check NEXT point NEXT inhibit*) OR cabozantinib OR cabometyx OR lenvatinib OR lenvima OR sorafenib OR nexavar OR sunitinib OR sutent OR erlotinib OR tarceva OR doxorubicin):ti
#23	#19 OR #20 OR #21 OR #22
#24	#18 OR #23
#25	#11 AND #24
#26	#11 AND #24 with Cochrane Library publication date Between Dec 2019 and May 2022
