# Supplementary material for: Utilising systematic reviews to assess potential overtreatment and claim for better evidence-based research: an analysis of anticancer drugs versus supportive care in advanced esophageal cancer
Source: Syst Rev. 2024 Jul 18;13:186. doi: 10.1186/s13643-024-02594-1 (PMC11256491; doi:10.1186/s13643-024-02594-1)
Supplement: Supplementary file 3 — Additional file 3: RoB assessment example. [file 13643_2024_2594_MOESM3_ESM.pdf]

| | | **RISK OF BIAS ASSESSMENT** | | | | | | | | | | | |
|---|---|---|---|---|---|---|---|---|---|---|---|---|---|
| | | Selection bias | | | | Performance bias | | Detection bias | | Attrition bias | | Reporting bias | |
| **Reviewer** | **Study ID (automatic, do** | Random sequence | Rationale | Allocation concealment | Rationale | Blinding of participants and | Rationale (specify if | Blinding of outcome | Rationale (specify if | Incomplete outcome data | Rationale | Selective reporting | Rationale |
| LL | **Fuchs 2014** | Low | Patients were randomly assigned in a 2:1 ratio, via a centralised interactive voice-response system | Low | Patients, medical and ancillary staff , the study investigators, and the sponsor were masked to group assignment. | Low | Double-blind | Low | Assessments (at least some of them) were carried out by the investigators, who were masked to group assigment. "Radiographically confirmed disease progression (investigator assessed by RECIST15)", "Investigator-assessed objective response rate was measured according to RECIST;15", | Low | 2 participants from each group did not receive the study group. 7 participants from the ramucirumab arm and 2 participants from the placebo arm withdrew consent. No information is provided about the participants lost to follow-up | Low | Results for the primary outcomes and for all secondary outcomes are provided in the manuscript. |
