# Supplementary material for: Utilising systematic reviews to assess potential overtreatment and claim for better evidence-based research: an analysis of anticancer drugs versus supportive care in advanced esophageal cancer
Source: Syst Rev. 2024 Jul 18;13:186. doi: 10.1186/s13643-024-02594-1 (PMC11256491; doi:10.1186/s13643-024-02594-1)

Overall survival (OS) outcome: Sensitivity analyses and meta-analyses (continuous, 6, 12 and 18-months)

OS sensitivity analysis (excluding study with high risk of bias –Ford 2014–)

Study or Subgroup	log[Hazard Ratio]	SE	Experimental Total	Control Total	Weight	Hazard Ratio IV, Random, 95% CI
**1.2.1 Targeted therapy**						
Dutton 2014 (COG)	-0.1054	0.0999	224	225	12.0%	0.90 [0.74, 1.09]
Fuchs 2014	-0.2536	0.1287	238	117	9.1%	0.78 [0.60, 1.00]
Huang 2021 (ALTER1102)	0.1655	0.2047	109	55	4.7%	1.18 [0.79, 1.76]
Kang 2019 (ANGEL)	-0.0726	0.1166	308	152	10.2%	0.93 [0.74, 1.17]
Li 2016	-0.3439	0.1418	176	91	8.0%	0.71 [0.54, 0.94]
Ohtsu 2013 (GRANITE-1)	-0.1054	0.093	439	217	12.8%	0.90 [0.75, 1.08]
Pavlakis 2016 (INTEGRATE)	-0.3011	0.1899	97	50	5.3%	0.74 [0.51, 1.07]
**Subtotal (95% CI)**			**1591**	**907**	**62.2%**	**0.87 [0.79, 0.95]**
Heterogeneity: Tau² = 0.00; Chi² = 6.38, df = 6 (P = 0.38); I² = 6%						
Test for overall effect: Z = 2.96 (P = 0.003)						
**1.2.2 Immunotherapy**						
Kang 2017 (ONO-4538-12, ATTRACTION-2)	-0.478	0.0996	330	163	12.0%	0.62 [0.51, 0.75]
**Subtotal (95% CI)**			**330**	**163**	**12.0%**	**0.62 [0.51, 0.75]**
Heterogeneity: Not applicable						
Test for overall effect: Z = 4.80 (P < 0.00001)						
**1.2.3 Chemotherapy**						
Hall 2021 (GO2)	-0.3711	0.3463	23	22	1.9%	0.69 [0.35, 1.36]
Levard 1998	-0.2744	0.1039	48	46	11.5%	0.76 [0.62, 0.93]
Nicolau 1982	-0.1625	0.4675	12	12	1.1%	0.85 [0.34, 2.13]
Shitara 2018 (TAGS)	-0.3711	0.1065	337	170	11.3%	0.69 [0.56, 0.85]
**Subtotal (95% CI)**			**420**	**250**	**25.8%**	**0.73 [0.63, 0.84]**
Heterogeneity: Tau² = 0.00; Chi² = 0.56, df = 3 (P = 0.91); I² = 0%						
Test for overall effect: Z = 4.45 (P < 0.00001)						
**Total (95% CI)**			**2341**	**1320**	**100.0%**	**0.79 [0.72, 0.87]**
Heterogeneity: Tau² = 0.01; Chi² = 18.03, df = 11 (P = 0.08); I² = 39%						
Test for overall effect: Z = 4.67 (P < 0.00001)						
Test for subgroup differences: Chi² = 10.73, df = 2 (P = 0.005), I² = 81.4%						

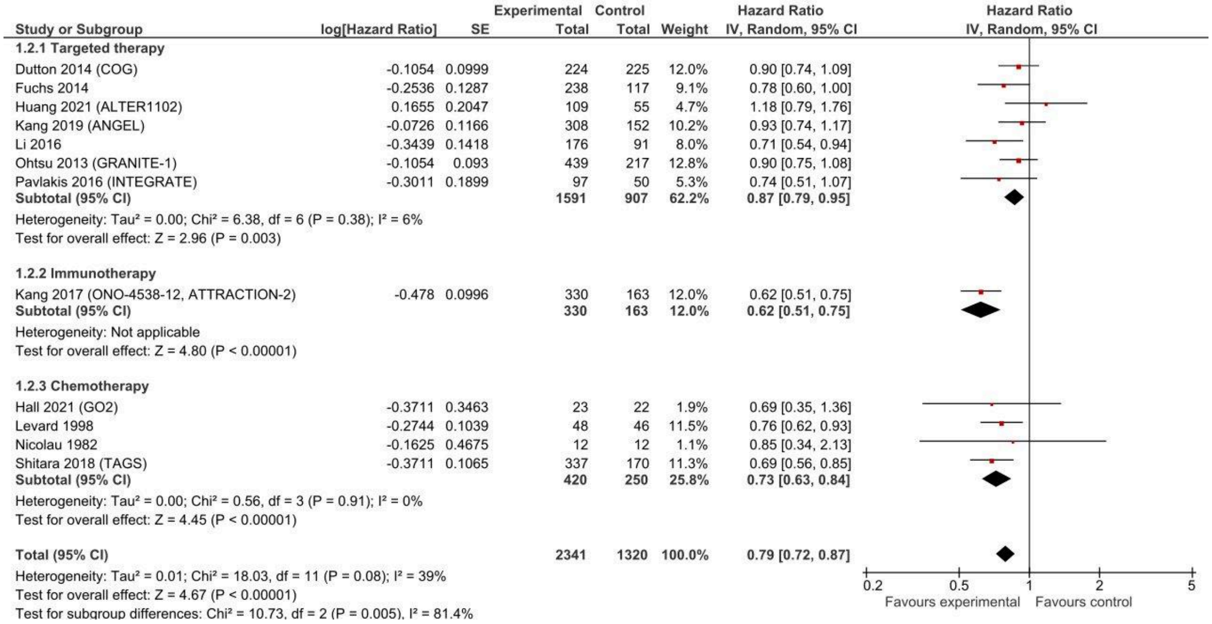

OS sensitivity analysis (excluding studies considering gastric cancer)

Study or Subgroup	log[Hazard Ratio]	SE	Experimental Total	Control Total	Weight	Hazard Ratio IV, Random, 95% CI
**1.1.1 Targeted therapy**						
Dutton 2014 (COG)	-0.1054	0.0999	224	225	41.9%	0.90 [0.74, 1.09]
Fuchs 2014	-0.2536	0.1287	238	117	0.0%	0.78 [0.60, 1.00]
Huang 2021 (ALTER1102)	0.1655	0.2047	109	55	14.8%	1.18 [0.79, 1.76]
Kang 2019 (ANGEL)	-0.0726	0.1166	308	152	0.0%	0.93 [0.74, 1.17]
Li 2016	-0.3439	0.1418	176	91	0.0%	0.71 [0.54, 0.94]
Ohtsu 2013 (GRANITE-1)	-0.1054	0.093	439	217	0.0%	0.90 [0.75, 1.08]
Pavlakis 2016 (INTEGRATE)	-0.3011	0.1899	97	50	0.0%	0.74 [0.51, 1.07]
**Subtotal (95% CI)**			**333**	**280**	**56.7%**	**0.97 [0.76, 1.23]**
Heterogeneity: Tau² = 0.01; Chi² = 1.41, df = 1 (P = 0.23); I² = 29%						
Test for overall effect: Z = 0.24 (P = 0.81)						
**1.1.2 Immunotherapy**						
Kang 2017 (ONO-4538-12, ATTRACTION-2)	-0.478	0.0996	330	163	0.0%	0.62 [0.51, 0.75]
**Subtotal (95% CI)**			**0**	**0**		**Not estimable**
Heterogeneity: Not applicable						
Test for overall effect: Not applicable						
**1.1.3 Chemotherapy**						
Ford 2014 (COUGAR-02)	-0.4005	0.1596	84	84	0.0%	0.67 [0.49, 0.92]
Hall 2021 (GO2)	-0.3711	0.3463	23	22	0.0%	0.69 [0.35, 1.36]
Levard 1998	-0.2744	0.1039	48	46	40.0%	0.76 [0.62, 0.93]
Nicolau 1982	-0.1625	0.4675	12	12	3.2%	0.85 [0.34, 2.13]
Shitara 2018 (TAGS)	-0.3711	0.1065	337	170	0.0%	0.69 [0.56, 0.85]
**Subtotal (95% CI)**			**60**	**58**	**43.3%**	**0.76 [0.63, 0.93]**
Heterogeneity: Tau² = 0.00; Chi² = 0.05, df = 1 (P = 0.82); I² = 0%						
Test for overall effect: Z = 2.65 (P = 0.008)						
**Total (95% CI)**			**393**	**338**	**100.0%**	**0.87 [0.74, 1.03]**
Heterogeneity: Tau² = 0.01; Chi² = 4.01, df = 3 (P = 0.26); I² = 25%						
Test for overall effect: Z = 1.57 (P = 0.12)						
Test for subgroup differences: Chi² = 2.29, df = 1 (P = 0.13), I² = 56.4%						

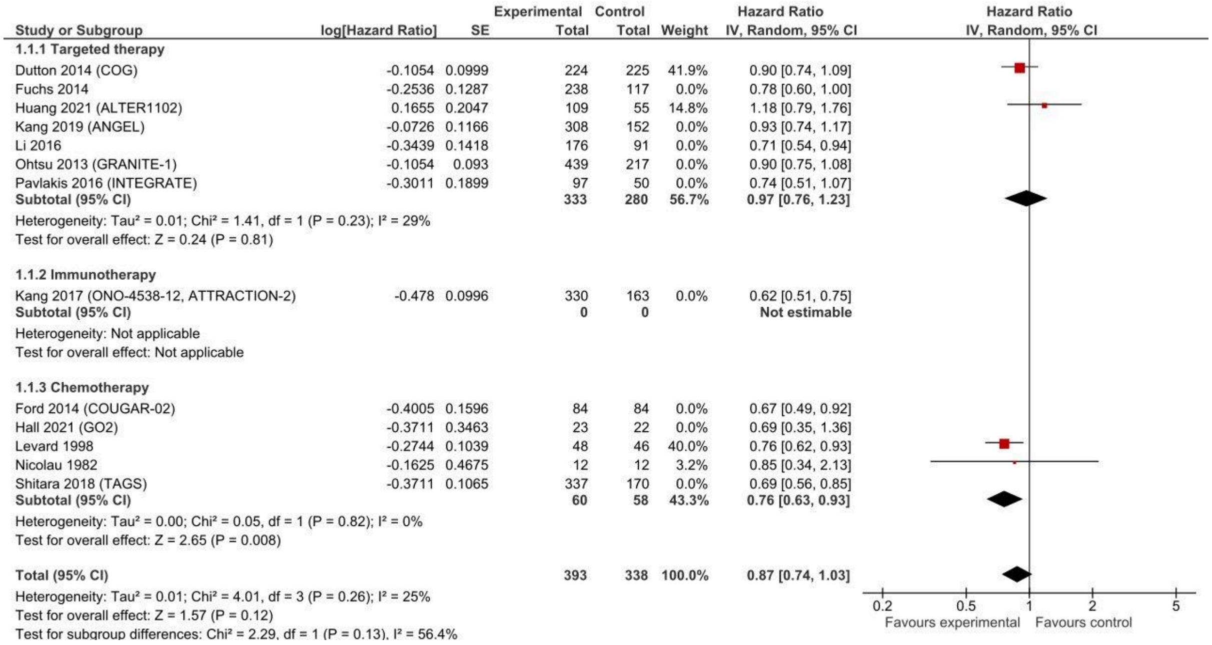

OS as continuous outcome

Study or Subgroup	Experimental Mean	Experimental SD	Experimental Total	Control Mean	Control SD	Control Total	Weight	Mean Difference IV, Random, 95% CI
**1.6.1 Targeted therapy**								
Alberts 1992	2.5315	1.7472	10	4.3726	1.7472	10	7.7%	-1.84 [-3.37, -0.31]
Dutton 2014 (COG)	3.73	3.7974	224	3.67	5.3283	225	12.1%	0.06 [-0.80, 0.92]
Fuchs 2014	5.2	10.26	238	3.8	7.29	117	6.2%	1.40 [-0.46, 3.26]
Huang 2021 (ALTER1102)	6.11	9.0067	109	7.2	8.7668	55	3.3%	-1.09 [-3.96, 1.78]
Kang 2019 (ANGEL)	5.82	4.9864	308	5.13	4.1183	152	12.0%	0.69 [-0.17, 1.55]
Li 2016	6.5	11.4273	176	4.7	5.2819	91	5.6%	1.80 [-0.21, 3.81]
Ohtsu 2013 (GRANITE-1)	5.4	6.3964	439	4.3	3.7369	217	12.6%	1.10 [0.32, 1.88]
Pavlakis 2016 (INTEGRATE)	5.8	6.9464	97	4.5	3.8706	50	6.7%	1.30 [-0.45, 3.05]
**Subtotal (95% CI)**			**1601**			**917**	**66.3%**	**0.49 [-0.23, 1.21]**
Heterogeneity: Tau² = 0.56; Chi² = 16.90, df = 7 (P = 0.02); I² = 59%								
Test for overall effect: Z = 1.33 (P = 0.18)								
**1.6.2 Immunotherapy**								
Kang 2017 (ONO-4538-12, ATTRACTION-2)	5.26	6.0947	330	4.14	4.655	163	11.2%	1.12 [0.15, 2.09]
**Subtotal (95% CI)**			**330**			**163**	**11.2%**	**1.12 [0.15, 2.09]**
Heterogeneity: Not applicable								
Test for overall effect: Z = 2.26 (P = 0.02)								
**1.6.3 Chemotherapy**								
Ford 2014 (COUGAR-02)	5.2	5.0688	84	3.6	1.3824	84	10.2%	1.60 [0.48, 2.72]
Schmid 1993	2.531	11.018	40	3.452	11.018	46	1.4%	-0.92 [-5.59, 3.75]
Shitara 2018 (TAGS)	5.7	8.3993	337	3.6	3.3024	170	10.9%	2.10 [1.08, 3.12]
**Subtotal (95% CI)**			**461**			**300**	**22.5%**	**1.80 [1.05, 2.55]**
Heterogeneity: Tau² = 0.00; Chi² = 1.76, df = 2 (P = 0.42); I² = 0%								
Test for overall effect: Z = 4.72 (P < 0.00001)								
**Total (95% CI)**			**2392**			**1380**	**100.0%**	**0.83 [0.24, 1.42]**
Heterogeneity: Tau² = 0.56; Chi² = 27.12, df = 11 (P = 0.004); I² = 59%								
Test for overall effect: Z = 2.76 (P = 0.006)								
Test for subgroup differences: Chi² = 6.09, df = 2 (P = 0.05), I² = 67.2%								

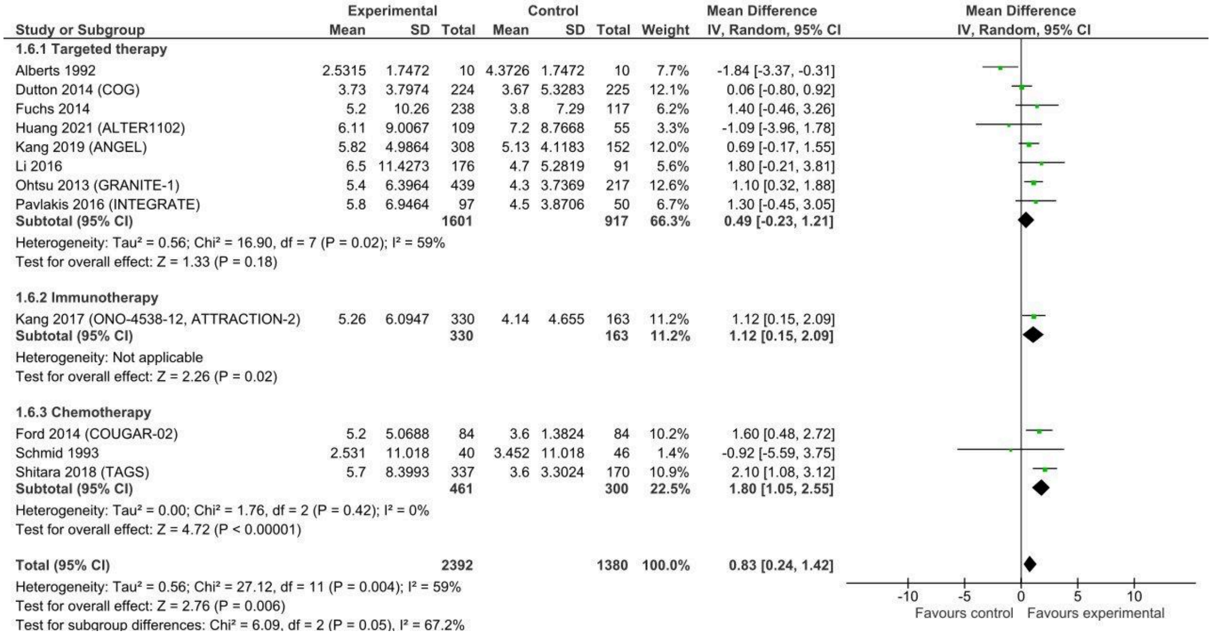

OS follow-up 6 months

Study or Subgroup	Experimental Events	Experimental Total	Control Events	Control Total	Weight	Risk Ratio M-H, Random, 95% CI
**1.7.1 Targeted therapy**						
Dutton 2014 (COG)	62	224	65	225	13.2%	0.96 [0.71, 1.29]
Fuchs 2014	92	238	34	117	11.6%	1.33 [0.96, 1.84]
Huang 2021 (ALTER1102)	42	109	26	55	9.9%	0.82 [0.57, 1.18]
Ohtsu 2013 (GRANITE-1)	195	439	82	217	19.6%	1.18 [0.96, 1.44]
Pavlakis 2016 (INTEGRATE)	39	97	14	50	6.0%	1.44 [0.87, 2.38]
**Subtotal (95% CI)**		**1107**		**664**	**60.4%**	**1.11 [0.93, 1.32]**
Total events	430		221			
Heterogeneity: Tau² = 0.01; Chi² = 6.20, df = 4 (P = 0.18); I² = 35%						
Test for overall effect: Z = 1.13 (P = 0.26)						
**1.7.2 Immunotherapy**						
Kang 2017 (ONO-4538-12, ATTRACTION-2)	143	330	54	163	15.9%	1.31 [1.02, 1.68]
**Subtotal (95% CI)**		**330**		**163**	**15.9%**	**1.31 [1.02, 1.68]**
Total events	143		54			
Heterogeneity: Not applicable						
Test for overall effect: Z = 2.10 (P = 0.04)						
**1.7.3 Chemotherapy**						
Ford 2014 (COUGAR-02)	33	84	19	84	6.6%	1.74 [1.08, 2.80]
Hall 2021 (GO2)	12	23	5	22	2.3%	2.30 [0.97, 5.45]
Nicolau 1982	4	12	2	12	0.8%	2.00 [0.45, 8.94]
Shitara 2018 (TAGS)	124	337	47	170	14.0%	1.33 [1.01, 1.76]
**Subtotal (95% CI)**		**456**		**288**	**23.8%**	**1.49 [1.18, 1.87]**
Total events	173		73			
Heterogeneity: Tau² = 0.00; Chi² = 2.13, df = 3 (P = 0.55); I² = 0%						
Test for overall effect: Z = 3.37 (P = 0.0007)						
**Total (95% CI)**		**1893**		**1115**	**100.0%**	**1.23 [1.07, 1.41]**
Total events	746		348			
Heterogeneity: Tau² = 0.01; Chi² = 13.33, df = 9 (P = 0.15); I² = 32%						
Test for overall effect: Z = 2.93 (P = 0.003)						
Test for subgroup differences: Chi² = 4.18, df = 2 (P = 0.12), I² = 52.1%						

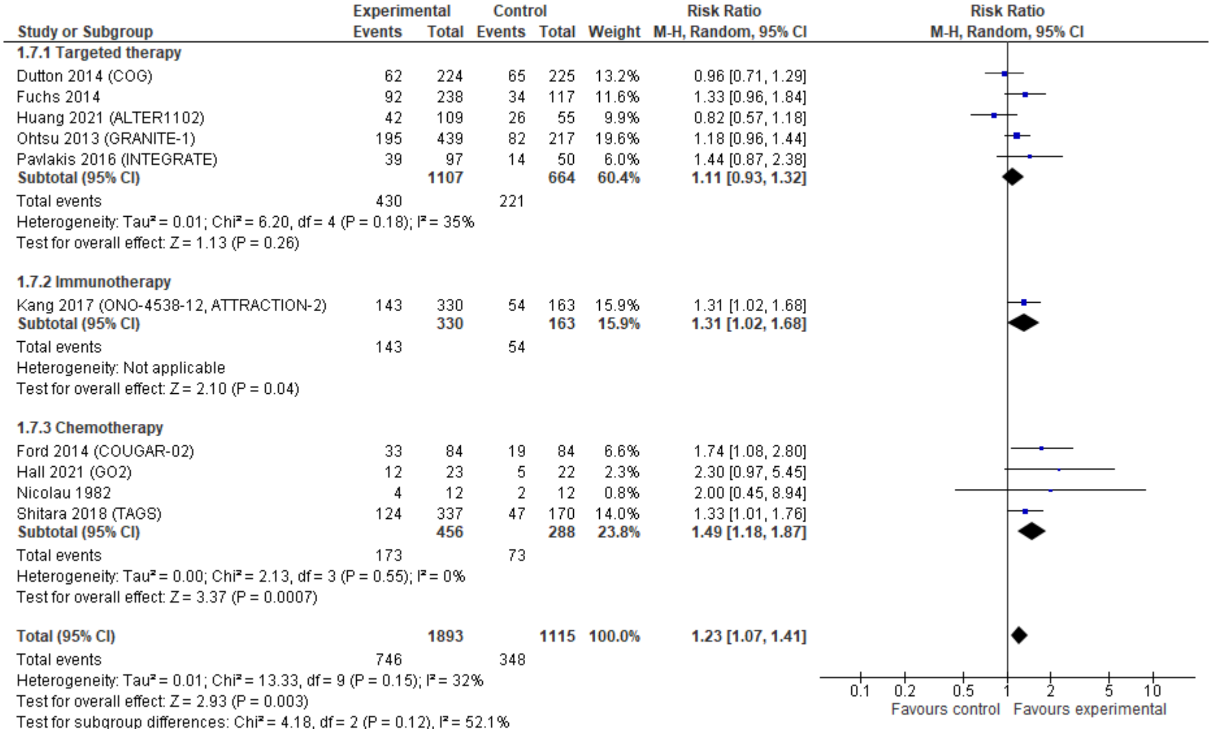

OS follow-up 12 months

Study or Subgroup	Experimental Events	Experimental Total	Control Events	Control Total	Weight	Risk Ratio M-H, Random, 95% CI
**1.8.1 Targeted therapy**						
Dutton 2014 (COG)	17	224	19	225	14.8%	0.90 [0.48, 1.68]
Fuchs 2014	17	238	7	117	11.4%	1.19 [0.51, 2.80]
Huang 2021 (ALTER1102)	11	109	11	55	12.6%	0.50 [0.23, 1.09]
Ohtsu 2013 (GRANITE-1)	52	439	28	217	18.1%	0.92 [0.60, 1.41]
**Subtotal (95% CI)**		**1010**		**614**	**56.9%**	**0.86 [0.64, 1.16]**
Total events	97		65			
Heterogeneity: Tau² = 0.00; Chi² = 2.52, df = 3 (P = 0.47); I² = 0%						
Test for overall effect: Z = 0.97 (P = 0.33)						
**1.8.2 Immunotherapy**						
Kang 2017 (ONO-4538-12, ATTRACTION-2)	84	330	18	163	17.4%	2.31 [1.44, 3.70]
**Subtotal (95% CI)**		**330**		**163**	**17.4%**	**2.31 [1.44, 3.70]**
Total events	84		18			
Heterogeneity: Not applicable						
Test for overall effect: Z = 3.46 (P = 0.0005)						
**1.8.3 Chemotherapy**						
Ford 2014 (COUGAR-02)	10	84	6	84	10.0%	1.67 [0.63, 4.38]
Hall 2021 (GO2)	6	23	0	22	1.9%	12.46 [0.74, 208.81]
Shitara 2018 (TAGS)	31	337	10	170	13.8%	1.56 [0.79, 3.11]
**Subtotal (95% CI)**		**444**		**276**	**25.8%**	**1.74 [0.99, 3.06]**
Total events	47		16			
Heterogeneity: Tau² = 0.01; Chi² = 2.06, df = 2 (P = 0.36); I² = 3%						
Test for overall effect: Z = 1.91 (P = 0.06)						
**Total (95% CI)**		**1784**		**1053**	**100.0%**	**1.23 [0.82, 1.86]**
Total events	228		99			
Heterogeneity: Tau² = 0.19; Chi² = 18.37, df = 7 (P = 0.01); I² = 62%						
Test for overall effect: Z = 1.00 (P = 0.32)						
Test for subgroup differences: Chi² = 13.56, df = 2 (P = 0.001), I² = 85.2%						

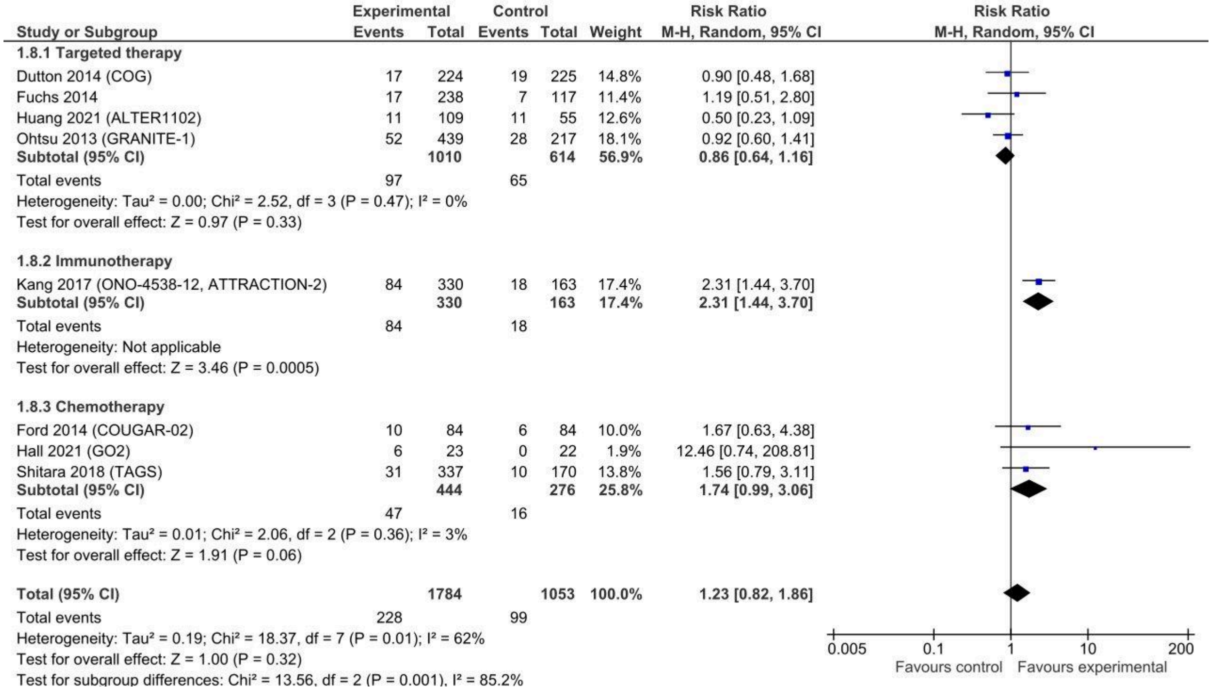

OS follow-up 18 months

Study or Subgroup	Experimental Events	Experimental Total	Control Events	Control Total	Weight	Risk Ratio M-H, Random, 95% CI
**1.9.1 Targeted therapy**						
Dutton 2014 (COG)	5	224	2	225	13.8%	2.51 [0.49, 12.81]
Fuchs 2014	3	238	2	117	12.8%	0.74 [0.12, 4.35]
Huang 2021 (ALTER1102)	7	109	6	55	18.0%	0.59 [0.21, 1.67]
Ohtsu 2013 (GRANITE-1)	6	439	8	217	17.9%	0.37 [0.13, 1.06]
**Subtotal (95% CI)**		**1010**		**614**	**62.4%**	**0.67 [0.32, 1.38]**
Total events	21		18			
Heterogeneity: Tau² = 0.12; Chi² = 3.83, df = 3 (P = 0.28); I² = 22%						
Test for overall effect: Z = 1.09 (P = 0.27)						
**1.9.2 Immunotherapy**						
Kang 2017 (ONO-4538-12, ATTRACTION-2)	51	330	6	163	19.5%	4.20 [1.84, 9.58]
**Subtotal (95% CI)**		**330**		**163**	**19.5%**	**4.20 [1.84, 9.58]**
Total events	51		6			
Heterogeneity: Not applicable						
Test for overall effect: Z = 3.41 (P = 0.0007)						
**1.9.3 Chemotherapy**						
Ford 2014 (COUGAR-02)	4	84	1	84	10.5%	4.00 [0.46, 35.04]
Shitara 2018 (TAGS)	7	337	0	170	7.6%	7.59 [0.44, 132.09]
**Subtotal (95% CI)**		**421**		**254**	**18.1%**	**5.06 [0.90, 28.47]**
Total events	11		1			
Heterogeneity: Tau² = 0.00; Chi² = 0.13, df = 1 (P = 0.72); I² = 0%						
Test for overall effect: Z = 1.84 (P = 0.07)						
**Total (95% CI)**		**1761**		**1031**	**100.0%**	**1.48 [0.57, 3.88]**
Total events	83		25			
Heterogeneity: Tau² = 1.06; Chi² = 19.73, df = 6 (P = 0.003); I² = 70%						
Test for overall effect: Z = 0.80 (P = 0.42)						
Test for subgroup differences: Chi² = 12.50, df = 2 (P = 0.002), I² = 84.0%						

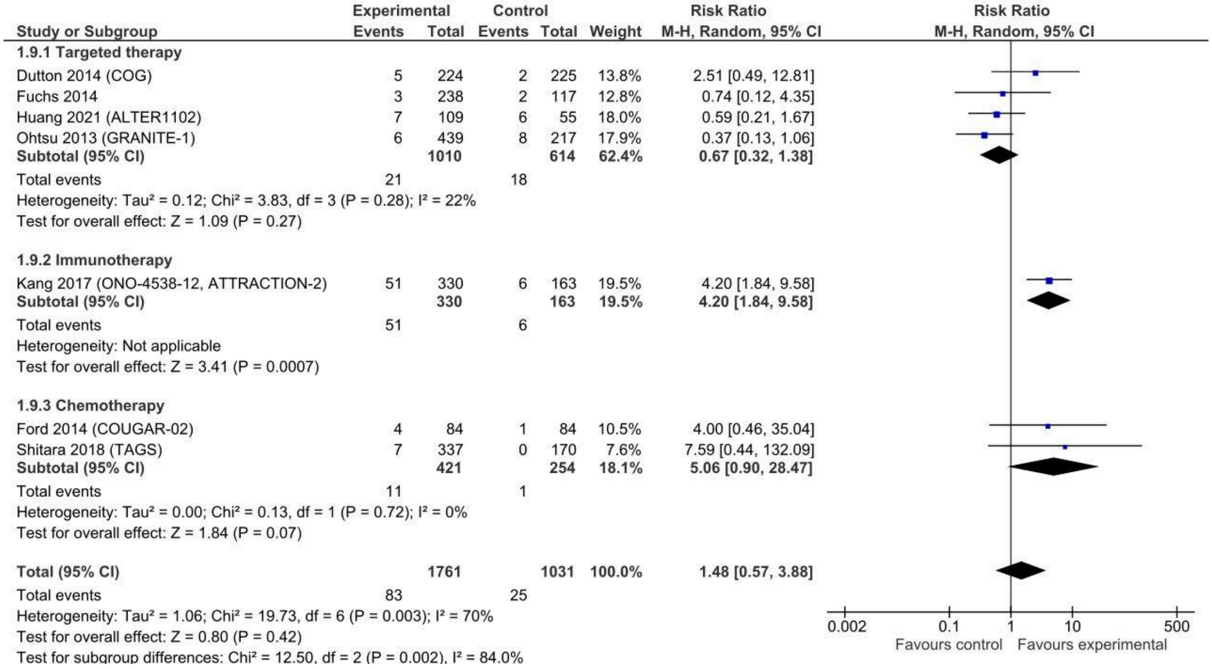

Supplement: Supplementary file 4 — Additional file 4: Overall survival (OS) outcome: Sensitivity analysis and meta-analyses (continuous, 6, 12, and 18 months). [file 13643_2024_2594_MOESM4_ESM.pdf]
