# Supplementary material for: Utilising systematic reviews to assess potential overtreatment and claim for better evidence-based research: an analysis of anticancer drugs versus supportive care in advanced esophageal cancer
Source: Syst Rev. 2024 Jul 18;13:186. doi: 10.1186/s13643-024-02594-1 (PMC11256491; doi:10.1186/s13643-024-02594-1)

Progression free survival (PFS) outcome: Sensitivity analysis and meta-analyses (continuous, 6, 12 and 18-months)

PFS sensitivity analysis (excluding studies considering gastric cancer)

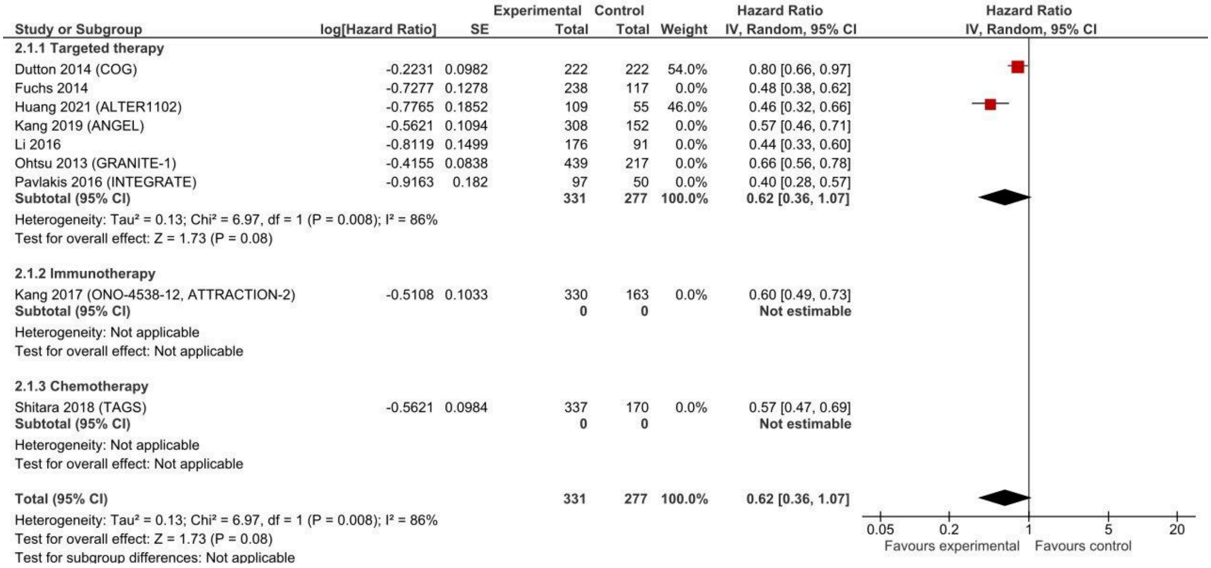

PFS as continuous outcome

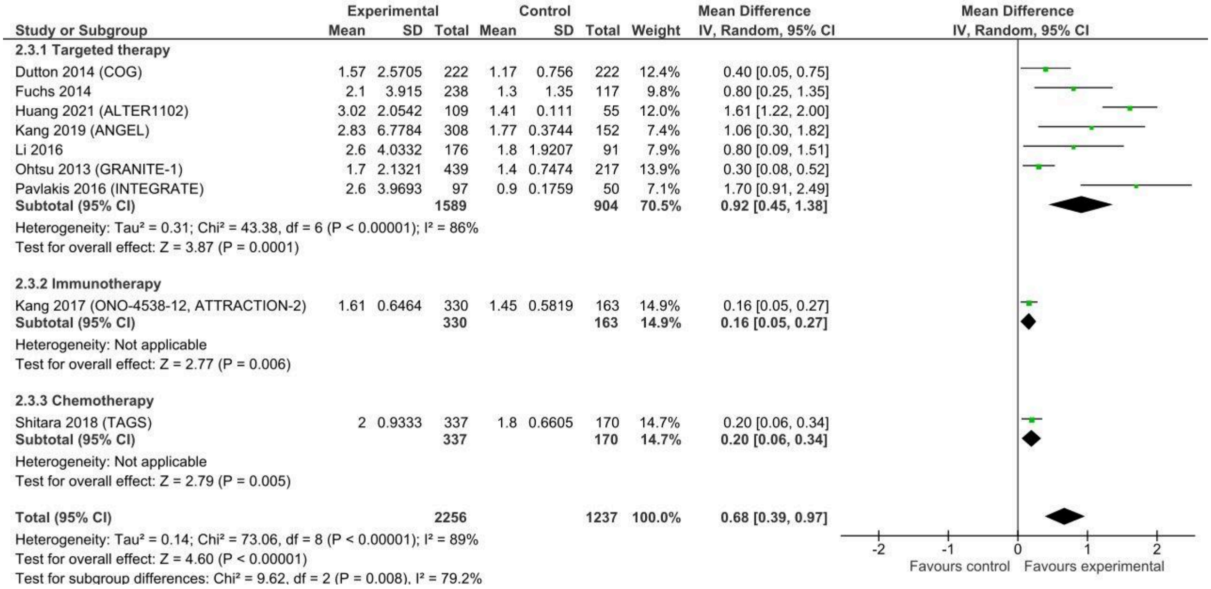

PFS follow-up 6 months

Study or Subgroup	Experimental Events	Experimental Total	Control Events	Control Total	Weight	Risk Ratio M-H, Random, 95% CI
**2.4.1 Targeted therapy**						
Dutton 2014 (COG)	25	222	20	222	21.6%	1.25 [0.72, 2.18]
Fuchs 2014	30	238	2	117	7.5%	7.37 [1.79, 30.33]
Huang 2021 (ALTER1102)	14	109	6	55	13.9%	1.18 [0.48, 2.90]
Ohtsu 2013 (GRANITE-1)	44	439	8	217	17.2%	2.72 [1.30, 5.67]
Pavlakis 2016 (INTEGRATE)	13	97	1	50	4.2%	6.70 [0.90, 49.77]
**Subtotal (95% CI)**		**1105**		**661**	**64.5%**	**2.23 [1.14, 4.38]**
Total events	126		37			
Heterogeneity: Tau² = 0.32; Chi² = 9.93, df = 4 (P = 0.04); I² = 60%						
Test for overall effect: Z = 2.34 (P = 0.02)						
**2.4.2 Immunotherapy**						
Kang 2017 (ONO-4538-12, ATTRACTION-2)	54	330	9	163	18.5%	2.96 [1.50, 5.85]
**Subtotal (95% CI)**		**330**		**163**	**18.5%**	**2.96 [1.50, 5.85]**
Total events	54		9			
Heterogeneity: Not applicable						
Test for overall effect: Z = 3.13 (P = 0.002)						
**2.4.3 Chemotherapy**						
Shitara 2018 (TAGS)	37	337	8	170	17.1%	2.33 [1.11, 4.90]
**Subtotal (95% CI)**		**337**		**170**	**17.1%**	**2.33 [1.11, 4.90]**
Total events	37		8			
Heterogeneity: Not applicable						
Test for overall effect: Z = 2.24 (P = 0.03)						
**Total (95% CI)**		**1772**		**994**	**100.0%**	**2.27 [1.46, 3.53]**
Total events	217		54			
Heterogeneity: Tau² = 0.15; Chi² = 11.28, df = 6 (P = 0.08); I² = 47%						
Test for overall effect: Z = 3.63 (P = 0.0003)						
Test for subgroup differences: Chi² = 0.38, df = 2 (P = 0.83), I² = 0%						

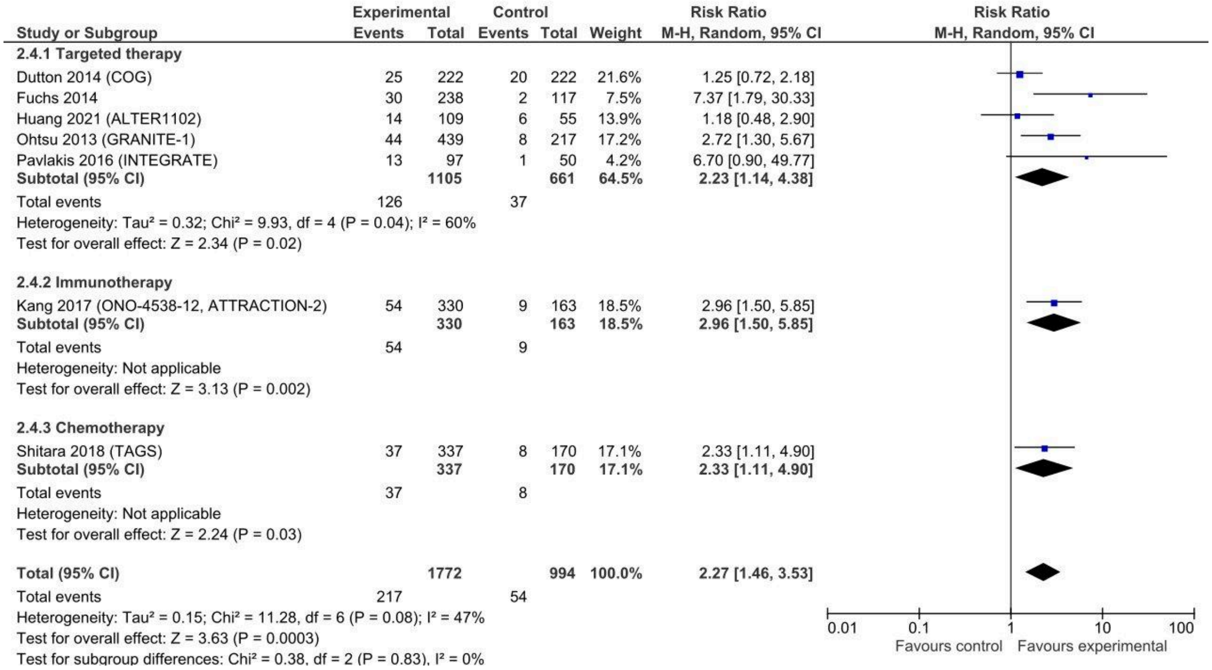

PFS follow-up 12 months

Study or Subgroup	Experimental Events	Experimental Total	Control Events	Control Total	Weight	Risk Ratio M-H, Random, 95% CI
**2.5.1 Targeted therapy**						
Dutton 2014 (COG)	8	222	3	222	29.6%	2.67 [0.72, 9.92]
Fuchs 2014	2	238	1	117	8.9%	0.98 [0.09, 10.73]
Huang 2021 (ALTER1102)	2	109	0	55	5.6%	2.55 [0.12, 52.12]
Ohtsu 2013 (GRANITE-1)	6	439	2	217	20.2%	1.48 [0.30, 7.29]
**Subtotal (95% CI)**		**1008**		**611**	**64.3%**	**1.92 [0.79, 4.69]**
Total events	18		6			
Heterogeneity: Tau² = 0.00; Chi² = 0.68, df = 3 (P = 0.88); I² = 0%						
Test for overall effect: Z = 1.44 (P = 0.15)						
**2.5.2 Immunotherapy**						
Kang 2017 (ONO-4538-12, ATTRACTION-2)	24	330	2	163	25.0%	5.93 [1.42, 24.77]
**Subtotal (95% CI)**		**330**		**163**	**25.0%**	**5.93 [1.42, 24.77]**
Total events	24		2			
Heterogeneity: Not applicable						
Test for overall effect: Z = 2.44 (P = 0.01)						
**2.5.3 Chemotherapy**						
Shitara 2018 (TAGS)	4	337	1	170	10.7%	2.02 [0.23, 17.91]
**Subtotal (95% CI)**		**337**		**170**	**10.7%**	**2.02 [0.23, 17.91]**
Total events	4		1			
Heterogeneity: Not applicable						
Test for overall effect: Z = 0.63 (P = 0.53)						
**Total (95% CI)**		**1675**		**944**	**100.0%**	**2.56 [1.25, 5.23]**
Total events	46		9			
Heterogeneity: Tau² = 0.00; Chi² = 2.54, df = 5 (P = 0.77); I² = 0%						
Test for overall effect: Z = 2.58 (P = 0.010)						
Test for subgroup differences: Chi² = 1.76, df = 2 (P = 0.41), I² = 0%						

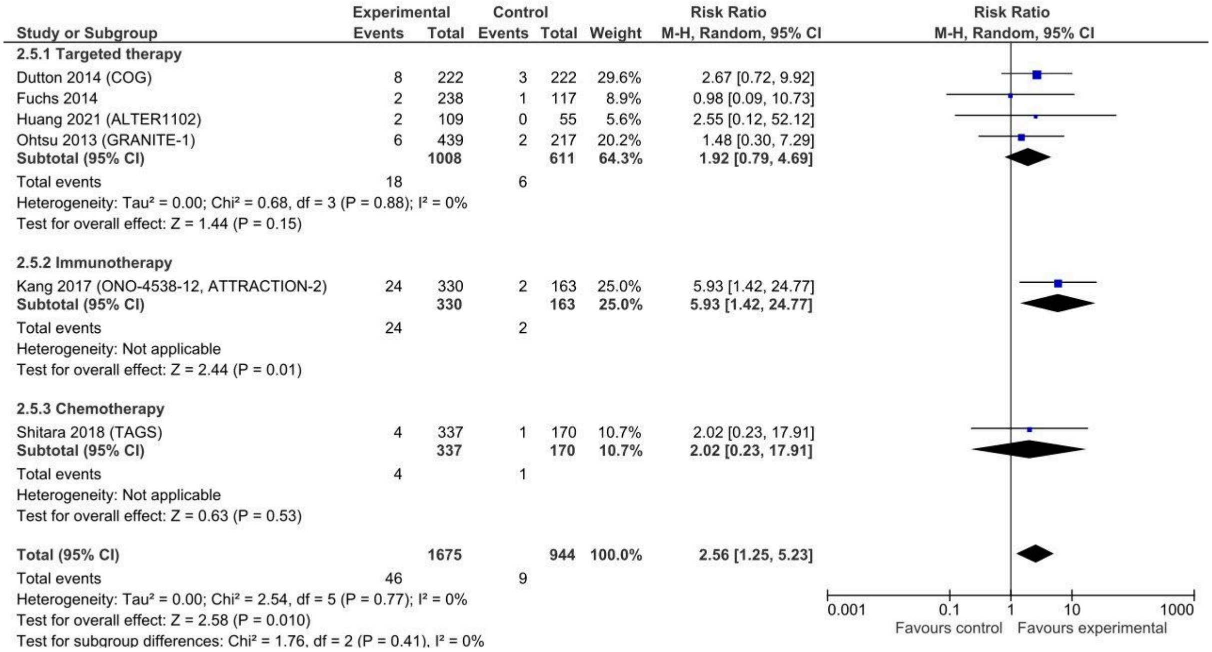

PFS follow-up 18 months

Study or Subgroup	Experimental Events	Experimental Total	Control Events	Control Total	Weight	Risk Ratio M-H, Random, 95% CI
**2.6.1 Targeted therapy**						
Dutton 2014 (COG)	3	222	0	222	20.7%	7.00 [0.36, 134.73]
Huang 2021 (ALTER1102)	2	109	0	55	20.1%	2.55 [0.12, 52.12]
Ohtsu 2013 (GRANITE-1)	1	439	2	217	26.9%	0.25 [0.02, 2.71]
**Subtotal (95% CI)**		**770**		**494**	**67.8%**	**1.39 [0.18, 11.05]**
Total events	6		2			
Heterogeneity: $Tau^2 = 1.35$; $Chi^2 = 3.35$, df = 2 (P = 0.19); $I^2 = 40\%$						
Test for overall effect: Z = 0.31 (P = 0.75)						
**2.6.2 Immunotherapy**						
Kang 2017 (ONO-4538-12, ATTRACTION-2)	13	330	1	163	32.2%	6.42 [0.85, 48.66]
**Subtotal (95% CI)**		**330**		**163**	**32.2%**	**6.42 [0.85, 48.66]**
Total events	13		1			
Heterogeneity: Not applicable						
Test for overall effect: Z = 1.80 (P = 0.07)						
**Total (95% CI)**		**1100**		**657**	**100.0%**	**2.26 [0.44, 11.62]**
Total events	19		3			
Heterogeneity: $Tau^2 = 1.10$; $Chi^2 = 4.96$, df = 3 (P = 0.17); $I^2 = 40\%$						
Test for overall effect: Z = 0.97 (P = 0.33)						
Test for subgroup differences: $Chi^2 = 1.07$, df = 1 (P = 0.30), $I^2 = 6.6\%$						

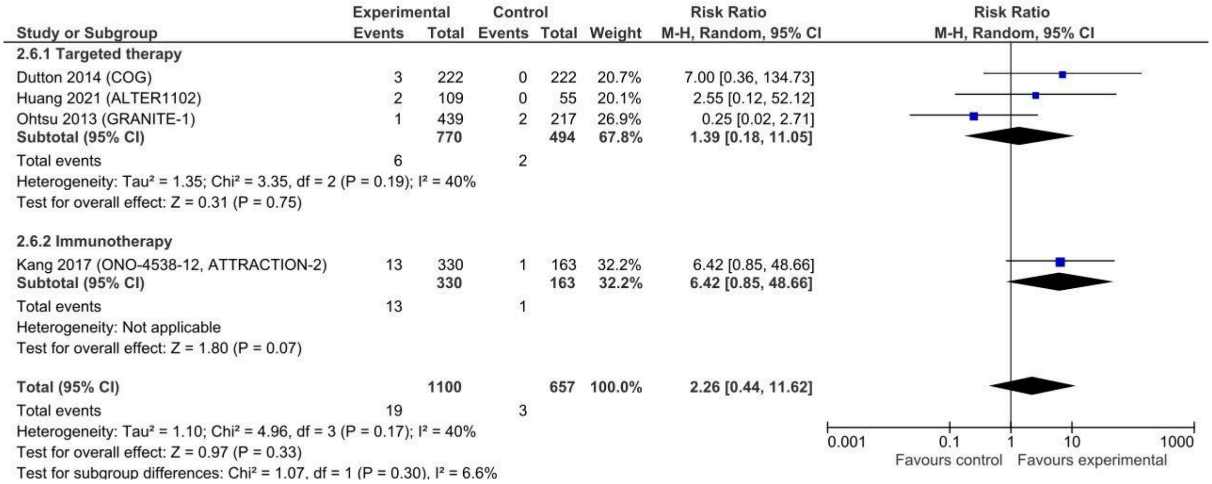

Supplement: Supplementary file 5 — Additional file 5: Progression-free survival (PFS) outcome: Sensitivity analysis and meta-analyses (continuous, 6, 12, and 18 months). [file 13643_2024_2594_MOESM5_ESM.pdf]
