# Supplementary material for: Utilising systematic reviews to assess potential overtreatment and claim for better evidence-based research: an analysis of anticancer drugs versus supportive care in advanced esophageal cancer
Source: Syst Rev. 2024 Jul 18;13:186. doi: 10.1186/s13643-024-02594-1 (PMC11256491; doi:10.1186/s13643-024-02594-1)
Supplement: Supplementary file 6 — Additional file 6: Quality of life (QoL) in studies comparing anticancer drugs to supportive care for advanced esophageal cancer. [file 13643_2024_2594_MOESM6_ESM.pdf]

Quality of life in studies comparing anticancer drugs to supportive care for advanced esophageal cancer

Study	Interventio n	Scales	Description	Conclusion
Dutton 2014	BIO/TT	EORTC QLQ-C30 ; EORTC QLQ-OG 25	treatment effect at 4 weeks for: Global QoL; QLQ-C30 function scores; QLQ-C30 and OG25 symptoms scores	difference adjusted mean Global QoL 2.69 (95% CI –2.33 to 7.72, n=231, p=0·293)
Ford 2014	CT	EORTC QLQ-C30 ; EORTC QLQ-STO 22	physical and social function and fatigue (QLQ-C30); eating restrictions and dysphagia (QLQ-STO22)	benefits for docetaxel were seen in all pre-specified important domains, of which dysphagia was statistically significant (p=0·02), and for several exploratory domains<br><br>patients in the docetaxel group reported less general pain (p=0·0008), abdominal pain (p=0·01), nausea and vomiting (p=0·02), and constipation (p=0·02) than those in the control group, but similar global HRQoL (p=0·53)
Fuchs 2014	BIO/TT	EORTC QLQ-C30	patient-reported global quality of life 6 weeks after start of treatment initiation	a larger proportion of those in the ramucirumab group reported stable or improved global quality of life than those in the placebo group (p=0·23)
Hall 2021	CT	EORTC QLQ-C30 ; EQ-5D-3 L	fatigue scale, global health status	both QL and fatigue were non significantly better with chemotherapy than BSC

Kang 2019	BIO/TT	EORTC QLQ-C30; EORTC QLQ-STO22; EQ-5D-5L	change from baseline in Global QoL (EORTC QLQ-C30); change from baseline in EORTC QLQ-STO22 Score; change from baseline in EQ-5D-5L VAS Score; number of participants per QoL dimension response as measured by EQ-5D-5L	NR
Li 2016	BIO/TT	EORTC QLQ-C30	QoL was assessed at baseline (7 days before first dose of study drug), after cycles two and three, and every two cycles thereafter until disease progression, death, or withdrawal of consent, whichever occurred first	no significant differences between the two groups at any time point with regard to QoL score for the different parameters of the EORTC QLQ-C30 (p >0.05)
Ohtsu 2013	BIO/TT	EORTC QLQ-C30	time to definitive 5% deterioration in the global health status/ QoL and physical, social, and emotional functioning scales	a trend for a slightly longer time to 5% deterioration in global QoL was observed for everolimus (median time to 5% deterioration, 1.51 months v 1.45 months; HR, 0.84; 95% CI, 0.69 to 1.03; P .094).<br><br>over time and versus placebo, everolimus recipients had higher mean scores for the global health status/QoL scale of the QLQ-C30 questionnaire

Pavlakis 2016	BIO/TT	EORTC QLQ-C30 ; EORTC QLQ-STO 2218; EQ-5D	QLQ-C30 Global Health Subscale mean estimates	for **r**egorafenib versus placebo were 53 (95% CI, 48 to 58) versus 58 (95% CI, 51 to 65) at week 4 and 54 (95% CI, 48 to 60) versus 56 (95% CI, 45 to 67) at week 8, respectively
Shitara 2018	CT	EORTC QLQ-C30 ; EORTC QLQ-ST0 22	mean QLQ-C30 Global Health Status (GHS) score, and subscale scores	QoL was maintained in TAGS, and there was a trend towards trifuridine/tipiracil reducing the risk of QoL deterioration compared with placebo
