# Supplementary material for: Utilising systematic reviews to assess potential overtreatment and claim for better evidence-based research: an analysis of anticancer drugs versus supportive care in advanced esophageal cancer
Source: Syst Rev. 2024 Jul 18;13:186. doi: 10.1186/s13643-024-02594-1 (PMC11256491; doi:10.1186/s13643-024-02594-1)
Supplement: Supplementary file 7 — Additional file 7: GRADE assessment and Summary of Findings (SoF) tables. [file 13643_2024_2594_MOESM7_ESM.pdf]

Data and analyses and summary of findings table for the main comparison

Comparison 1
Question: Chemotherapy compared to supportive care for advanced esophageal cancer

Certainty assessment							№ of patients		Effect		Certainty	Importance
№ of studies	Study design	Risk of bias	Inconsistency	Indirectness	Imprecision	Other considerations[e]	chemotherapy	supportive care	Relative (95% CI)	Absolute (95% CI)		
**Overall Survival (follow-up: range 12 months to 18 months)**												
5	randomised trials	not serious[a]	not serious	very serious[b]	not serious	none	504 participants	334 participants	**HR 0.72** (0.63 to 0.81) [death]	**109 more per 1,000** (from 55 more to 163 more)	⊕⊕◯◯ Low	
							-	10.0%				
**Progression free survival (follow-up: 12 months)**												
1	randomised trials	not serious	not serious	very serious[b]	not serious	none	337 participants	170 participants	**HR 0.57** (0.47 to 0.69) [death or progression]	**174 more per 1,000** (from 120 more to 222 more)	⊕⊕◯◯ Low	
							-	50.0%				
**Performance status (assessed with: ECOG)**												
1	randomised trials	not serious	not serious	very serious[b]	not serious	none	337 participants	170 participants	**HR 0.69** (0.56 to 0.85) [deterioration]	**129 more per 1,000** (from 55 more to 206 more)	⊕⊕◯◯ Low	
							-	20.0%				
**Toxicity**												
2	randomised trials	not serious[a]	not serious	very serious[b]	very serious[c]	none	284/416 (68.3%)	100/242 (41.3%)	**RR 2.36** (0.63 to 8.86)	**562 more per 1,000** (from 153 fewer to 1,000 more)	⊕◯◯◯ Very low	
**Quality of life**												
3	randomised trials	serious[d]	not serious	very serious[b]	not serious	none	Only one study showed significant results (high risk of bias). Significant improvement in quality of life was found with docetaxel in all pre-specified important domains, of which dysphagia was statistically significant (p=0·02), and for several exploratory domains.				⊕◯◯◯ Very low	

CI: confidence interval; **HR:** hazard Ratio; **RR:** risk ratio

Explanations

a. We did not downgrade even though Ford 2014 was assessed as high risk of bias (performance and detection) because a sensitivity analysis did not show differences
b. We downgraded two levels due to including GEJ and gastric population

c. Very wide confidence interval
d. We downgraded one level due to concerns about the high risk of performance bias.
e. Due to the low number of included studies informing each outcome (<10), we did not assess publication bias through a funnel plot.

Comparison 2
Question: Immunotherapy compared to supportive care for advanced esophageal cancer

Certainty assessment							№ of patients		Effect		Certainty	Importance
№ of studies	Study design	Risk of bias	Inconsistency	Indirectness	Imprecision	Other considerations[b]	immunotherapy	supportive care	Relative (95% CI)	Absolute (95% CI)		
**Overall survival**												
1	randomised trials	not serious	not serious	very serious[a]	not serious	none	330 participants	163 participants	**HR 0.62** (0.51 to 0.75) [death]	**140 more per 1,000** (from 78 more to 209 more)	⨁⨁◯◯ Low	
							-	10.0%				
**Progression free survival**												
1	randomised trials	not serious	not serious	very serious[a]	not serious	none	330 participants	163 participants	**HR 0.60** (0.49 to 0.73) [death or progression]	**160 more per 1,000** (from 103 more to 212 more)	⨁⨁◯◯ Low	
							-	50.0%				
**Toxicity**												
1	randomised trials	not serious	not serious	very serious[a]	not serious	none	39/330 (11.8%)	7/161 (4.3%)	**RR 2.72** (1.24 to 5.94)	**75 more per 1,000** (from 10 more to 215 more)	⨁⨁◯◯ Low	

CI: confidence interval; **HR:** hazard Ratio; **RR:** risk ratio

Explanations

a. We downgraded two levels due to including GEJ and gastric population
b. Due to the low number of included studies informing each outcome (<10), we did not assess publication bias through a funnel plot.

Comparison 3
Question: Biological targeted therapy compared to supportive care for advanced esophageal cancer

Certainty assessment							№ of patients		Effect		Certainty	Importance
№ of studies	Study design	Risk of bias	Inconsistency	Indirectness	Imprecision	Other considerations[c]	biological targeted therapy	supportive care	Relative (95% CI)	Absolute (95% CI)		
**Overall survival**												
7	randomised trials	not serious	not serious	very serious[a]	not serious	none	1591 participants	907 participants	**HR 0.87** (0.79 to 0.95) [death]	**35 more per 1,000** (from 12 more to 62 more)	⊕⊕◯◯ Low	
							-	10.0%				
**Progression free survival**												
7	randomised trials	not serious	not serious	very serious[a]	not serious	none	1589 participants	904 participants	**HR 0.55** (0.45 to 0.66) [death or recurrence]	**183 more per 1,000** (from 133 more to 232 more)	⊕⊕◯◯ Low	
							-	50.0%				
**Toxicity**												
5	randomised trials	not serious	not serious	very serious[a]	very serious[b]	none	653/1103 (59.2%)	303/660 (45.9%)	**RR 1.25** (1.01 to 1.54)	**115 more per 1,000** (from 5 more to 248 more)	⊕◯◯◯ Very low	
**Quality of life**												
6	randomised trials	not serious	not serious	very serious[a]	not serious	none	no significant differences between groups				⊕⊕◯◯ Low	

CI: confidence interval; **HR:** hazard Ratio; **RR:** risk ratio

Explanations

a. a. We downgraded two levels due to including GEJ and gastric population
b. Very wide confidence interval
c. Due to the low number of included studies informing each outcome (<10), we did not assess publication bias through a funnel plot.
